# Supplementary material for: Mathematical modeling links Wnt signaling to emergent patterns of metabolism in colon cancer
Source: Mol Syst Biol. 2017 Feb 9;13(2):912. doi: 10.15252/msb.20167386 (PMC5327728; doi:10.15252/msb.20167386)
Supplement: Supplementary file 1 — Appendix [file MSB-13-912-s001.pdf]

Appendix

Contents

A1 Image Processing . . . ii
A1.1 Description of image processing of experimental data . . . ii
A1.2 Description of overlay analysis from image processing data (Figure EV1) . . . ii
A1.3 Image processing parameters and data . . . iv
A1.4 Gradient of phospho-PDH spot density in SW480 xenograft tumors . . . v
A1.5 SW480 Mock xenograft tumor phospho-PDH immunohistochemistry . . . vii
A1.6 SW480 Mock xenograft tumor LEF1 immunohistochemistry . . . viii
A1.7 SW480 Mock xenograft tumor β-catenin immunohistochemistry . . . x
A1.8 SW480 dnLEF xenograft tumor phospho-PDH immunohistochemistry . . . xi
A1.9 SW480 dnLEF xenograft tumor β-catenin immunohistochemistry . . . xii
A1.10 Heterogeneity in SW480 dnTCF xenograft tumors . . . xiii
A1.11 SW480 dnTCF phospho-PDH immunohistochemistry . . . xiv
A1.12 SW480 dnTCF β-catenin immunohistochemistry . . . xv
A1.13 Statistical significance metabolic patterning . . . xvi
A1.14 Description of image processing of simulation results . . . xvii
A1.15 Quantification of IHC staining intensity and cells per spot in SW480 Mock and dnLEF pPDH staining . . . xviii
A2 Nondimensionalization . . . xix
A3 Wnt-PDK-Lactate-HIF1α Cross-Feeding Model . . . xxii
A3.1 Augmenting the Wnt Signaling Model . . . xxii
A3.2 Results . . . xxiv
A3.3 Discussion . . . xxvii
A3.4 Numerical method . . . xxvii
A4 Nutrient diffusion simulation . . . xxviii
A5 Parameter exploration . . . xxix
A6 Diffusive stability analysis . . . xxx
A7 Simulation of in vitro tumor growth with DCA and XAV939 treatment . . . xxxii
A7.1 Boundary Conditions . . . xxxii
A7.2 Equations . . . xxxii
A7.3 Parameter values for in vitro model . . . xxxiv
A7.4 Simulations of DCA and XAV939 treatment on in vitro tumors . . . xxxv
A8 Synergy is evident in simulations with combined DCA and XAV939 treatment . . . xxxvi
A9 Heterogeneity in orthotopic tumors . . . xxxviii
A10 Vessel density versus pPDH expression in relative to Mock tumor . . . xxxix

A1 Image Processing

A1.1 Description of image processing of experimental data

Image processing for spot contours was done using MATLAB. Below is a detailed description of the processing including the built-in MATLAB functions that were used (appended by .m). Thresholds for the MATLAB functions are given in Appendix Table S3.

1. Read the image into MATLAB and output an $n \times n \times 3$ matrix of RGB values using imread.m.
2. Manually choose one of the R, G, or B matrices. Convert this matrix to black and white (0's and 1's) using im2bw.m (setting the threshold manually); convert by subtracting this matrix from 1 so that white (a value of 1) is part of a spot and black (a value of 0) is not part of a spot.
3. Apply a noise filter with medfilt2.m (setting the threshold manually) to remove as many of the smaller spots as possible (this function is a median filter used to reduce "salt and pepper" noise).
4. Fill in any holes using imfill.m. Set any values in the output matrix of imfill from 0.5 to 1.
5. Use bwlabel.m to label connected components.
6. Use regionprops.m to get centroid locations and areas.
7. Define an area limit (setting the threshold manually) to plot only those spots whose area is larger than this limit.
8. Set a threshold for distance between any two centroids (in our case, we used 35 pixels, or approximately 11 μm, which we estimate to be the diameter of a cell). If any two centroids are within this distance, group all of these spots together and take the convex hull using convhull.m to visualize the spot. The centroid of the combined spots, or cluster of cells, is computed by taking the weighted average of the centroids, where weights are based on areas. The area of the combined spots is computed as the sum of the original areas.

In the following sections, images are shown with both red and blue contours. Red contours were found using the steps 1-7 above. Blue contours (convex hulls) were found using step 8 above.

A1.2 Description of overlay analysis from image processing data (Figure EV1)

Spot contour overlay using image processing was done using MATLAB. Below is a detailed description of how the overlay was formed using built-in MATLAB functions that were used (appended by .m). Thresholds for the MATLAB functions are given in Appendix Table S

1. Apply steps 1 and 2 from A1.1 for both pPDH and LEF1 experimental images to obtain two separate matrices M_{pPDH} and M_{LEF1}.
2. Form matrix $\overline{M}$ by multiplying M_{pPDH} by M_{LEF1} entry by entry (not matrix multiplication). Matrix $\overline{M}$ now contains overlapping regions of pPDH and LEF1 where white (a value of 1) is part of an overlapping region and black (a value of 0) is a non-overlapping region.
3. Form matrix $\overline{M}_{pPDH} = M_{pPDH} - \overline{M}$ and $\overline{M}_{LEF1} = M_{LEF1} - \overline{M}$ (to remove overlapping regions). Now, $\overline{M}_{pPDH}$, $\overline{M}_{LEF1}$ and $\overline{M}$ are matrices that do not overlap where the value of 1 is located. Form matrix $\overline{S} = 1 * \overline{M}_{pPDH} + 2 * \overline{M}_{LEF1} + 3 * \overline{M}$, so that each a value of 0 assigned in non-spot region, a value of 1 is assigned in pPDH spots (minus the overlap), a value of 2 is assigned in LEF1 spots (minus the overlap), a value of 3 is assigned in the overlapping region.
4. Use imagesc.m to visualize overlay with matrix $\overline{S}$ using colormap.m (setting custom colors by manually selecting a 4 by 3 matrix where each row represents a color for the numbers 0, 1, 2, 3).

Percent coverage. The 4x (leftmost) images in Figs. 1A and 1B are both the same size and the images were assumed to be lined up as close as possible. In each image, a pixel inside a red contour was considered positively stained and a pixel outside a red contour was considered negatively stained. In the Mock pPDH image in Fig. 1A, there are 101,712 pixels inside the contours and there are 479,314 pixels in the tumor section, which yields a 21.2% coverage of pPDH spots. In the Mock LEF-1 image in Fig. 1B, there are 97,368 pixels inside the contours and there are 481,623 pixels in the tumor section, which yields a 20.2% coverage of LEF-1 spots. The number of pixels in the tumors in the images in Figs. 1A and 1B are slightly different because these images are serial sections. Because they are close, we can approximately pair the pixels in the images by assuming that each pixel location in one slice corresponds to the same pixel location in the other slice. The staining in each pixel pair can be described using a $2x2$ contingency matrix. For example, when there is both pPDH and LEF1 staining in the pixel pair, the contingency matrix is given in Table S 1. The contingency matrices in the other cases (e.g., pPDH positive stain/LEF1 negative stain, etc) correspond to permutations of the location of the "1" in the matrix entries.

Appendix Table S 1: $2x2$ contingency matrix showing LEF1 and pPDH staining in a pixel pair

	**pPDH**	**no pPDH**
**LEF1**	1	0
**no LEF1**	0	0

The number of pixels that overlap between the LEF-1 and pPDH images is 35,503 (blue area in Fig. EV1). Thus, the percent overlap of pPDH in LEF-1 spots is $35,503/97,368 = 36.4\%$ and the percent overlap of LEF-1 in pPDH spots is $35,503/101,712 = 34.9\%$. The area fraction of overlap between LEF-1 and pPDH spots in the tumor sections are $35,503/481,623 = 7.4\%$ and $35,503/479,314 = 7.4\%$, respectively.

Cochran-Mantel-Haenszel test. To test whether there is an association between the pPDH and the LEF1 spots, we used the Cochran-Mantel-Haenszel test (Cochran, 1954; Mantel & Haenszel, 1959). By collapsing the contingency matrices for all the pixels in the images, we obtain a single 2 x 2 contingency table that describes the numbers of pixel pairs with each type of staining. For Fig. 1B, this gives Table S 2

Appendix Table S 2: Collapsed 2 x 2 contingency matrix for all pixel pairs

	**pPDH**	**no pPDH**	**Total**
**LEF1**	35503	61865	97368
**no LEF1**	66209	318046	384255
**Total**	101712	379911	481623

The null hypothesis is that the pPDH and LEF1 spots are conditionally independent. Then, $z^2 = (n_{21} - n_{12})^2/(n_{21} + n_{22})$, where n_{21} and n_{12} are the off-diagonal terms in the contingency matrix, is a χ^2 statistic with 1 degree of freedom. Calculating, we obtain $z^2 = 147$ from which we conclude $p < 0.0001$. This allows us to reject the null hypothesis and we conclude that pPDH and LEF1 spots are significantly associated with each other. The results obtained using Fig. 1A are similar.

A1.3 Image processing parameters and data

Appendix Table S 3: **Parameters and resulting number of spots in image processing of IHC stains**

Sample	RGB	im2bw	medfilt2	Minimum area	Number of spots
Mock pPDH 1	B	0.3	15	700	98
Mock pPDH 2	B	0.28	10	700	75
Mock pPDH 3	B	0.4	15	700	89
mock LEF1 1	G	0.55	10	800	69
mock LEF1 2	B	0.3	15	500	77
mock LEF1 3	B	0.39	15	500	92
mock LEF1 4	B	0.3	15	500	87
mock LEF1 5	B	0.3	15	500	100
mock β-catenin 1	G	0.62	10	200	301
mock β-catenin 2	G	0.62	10	200	315
mock β-catenin 3	G	0.62	10	200	272
dnLEF pPDH 1	B	0.5	18	1500	29
dnLEF pPDH 2	B	0.5	18	1500	35
dnLEF pPDH 3	B	0.5	18	1500	24
dnLEF pPDH 4	B	0.55	20	1500	37
dnLEF β-catenin	B	0.35	10	1000	74
dnTCF pPDH 1	B	0.35	17	1500	31
dnTCF pPDH 2	B	0.33	20	1500	33
dnTCF pPDH 3	B	0.28	20	1500	44
dnTCF β-catenin 1	B	0.45	15	2000	35
dnTCF β-catenin 2	B	0.45	15	2000	55
dnTCF β-catenin 3	B	0.45	15	2000	26

Appendix Table S 4: **Parameters and resulting number of spots in image processing of IHC stains for overlay analysis**

Sample	RGB	im2bw	medfilt2	Minimum area	Number of spots
Mock pPDH 1	B	0.325	10	140	131
Mock pPDH 2	B	0.39	10	140	106
Mock pPDH 3	B	0.36	10	140	153
Mock LEF1 1	B	0.3	10	100	173
Mock LEF1 2	B	0.4	10	100	166
Mock LEF1 3	B	0.4	10	100	191

Parameter values and resulting number of spots in image analysis of pPDH, LEF1, and β-catenin stains. The terms im2bw and medfilt2 refer to built-in Matlab tools. Minimum area refers to the smallest area (in terms of square pixels) that were outlined. Number of spots refers to number of resulting spots outlined from the image processing, after combining spots if their centroids are within some distance (see image processing description in previous section). Thresholds for image analysis were set by user input to capture strongest signals from the epithelial part of the tumors, avoiding mouse stroma and vessels.

A1.4 Gradient of phospho-PDH spot density in SW480 xenograft tumors

We quantified and visualized the changes in spot densities from the tumor periphery to the core using Matlab and ImageJ tools. To identify contours of the spots, we applied steps 1-7 as in A1.1. Several large artificial "spots" were formed on the periphery when only one parameter set was used for the entire image (due to changes in intensity from the periphery (left) to the core (right) in the tumor). Thus, we separately analyzed the two largest "spots" near the periphery and formed contours of the spots using different parameters. We replaced the large "spots" with the corresponding new contours. As indicated in figure 1, we divided the image into 8 bins from left to right. For each bin we computed the total area of the spots. If the bin divides a spot into multiple pieces, the areas of each individual piece contained within the bin were added and the total areas of the spots in each bin were plotted in (C). To visualize the gradient in spot density and intensity, we used the ImageJ plugin "Interactive 3D Surface Plot" to create topographic maps from the green channel of tumor images (D-G). The spikes at the right are from tears in the tissue.

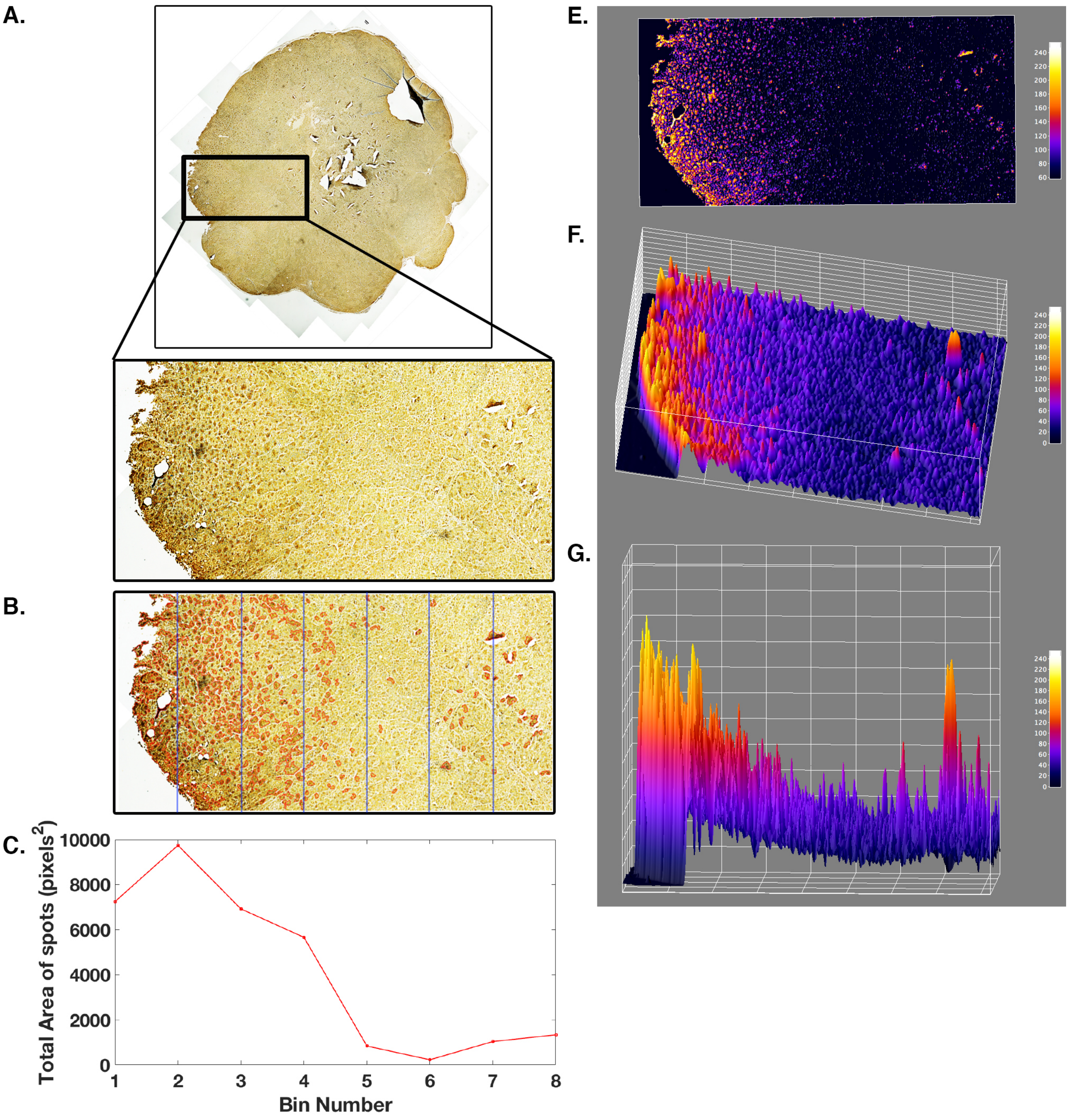

Appendix Figure S 1: Gradient of phospho-PDH spot density in SW480 xenograft tumors (A) A section of the pPDH-stained SW480 xenograft tumors was selected for analysis. (B) We divided the image into 8 bins (1 is leftmost and 8 is rightmost). For each bin we computed the total area of the spots. If the bin divides a spot into multiple pieces, the areas of each individual piece contained within the bin were added. (C) The areas of the spots in each bin are plotted and show that the areas of the spots generally decreases towards the core of the tumor. (D-F) To further visualize the gradient in spot density and intensity, the ImageJ plugin "Interactive 3D Surface Plot" was used to create topographic maps from the green channel of tumor images. (D) Top view, showing ability to detect spots using ImageJ. (F) Isometric view (G) Side view, showing intensity of spotting decreases towards the core of the tumor. The spikes at the right are from tears in the tissue.

A1.5 SW480 Mock xenograft tumor phospho-PDH immunohistochemistry

The figures that follow are stains that were processed and whose data (area of each spot and distance to nearest neighbor) appear in the image analysis scatter plots in the main text (Figure 1C).

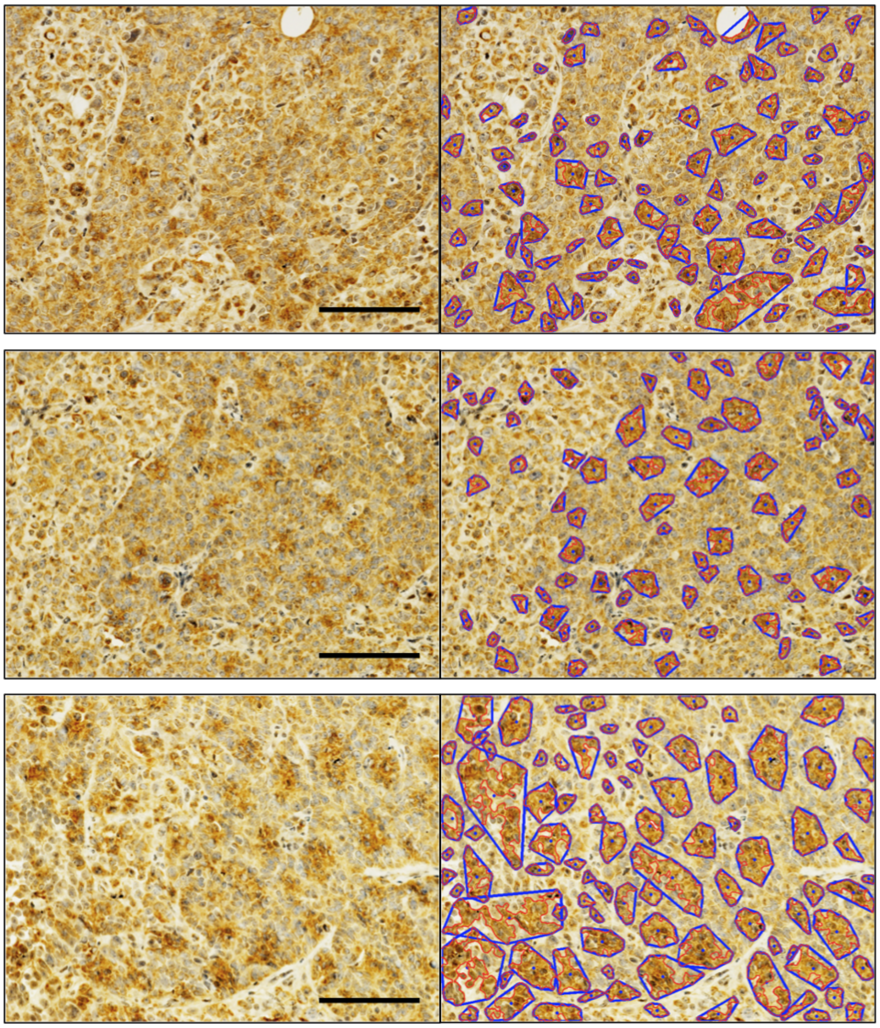

Appendix Figure S 2: Mock phospho-PDH contours. Scalebars indicate 100μm, with each images' dimensions approximately $440 \times 330 \mu m^2$. The left column shows stained images. The right column shows stained images with red contour outlines drawn around each spot and blue convex hull outlines which group red spots together if their centroids are within 35 pixels (approximately 11 μm) of each other (see image processing methods, A1.1).

A1.6 SW480 Mock xenograft tumor LEF1 immunohistochemistry

The figures that follow are stains that were processed and whose data (area of each spot and distance to nearest neighbor) appear in the image analysis scatter plots in the main text (Figure 1C).

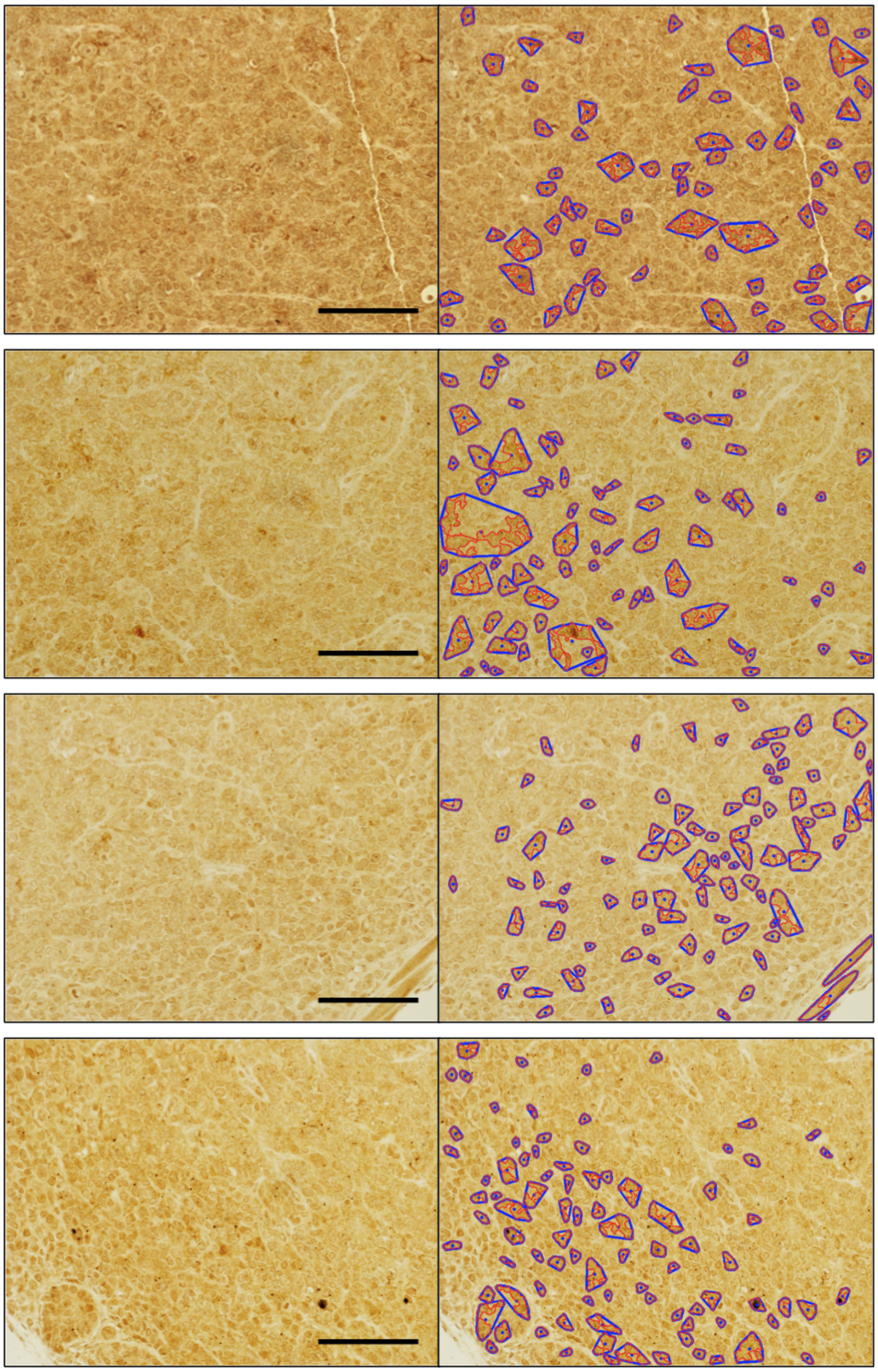

Appendix Figure S 3: Mock LEF1 contours. Scalebars indicate 100μm, with each images' dimensions approximately 440 $\times$ 330μm^2. The left column shows stained images. The right column shows stained images with red contour outlines drawn around each spot and blue convex hull outlines which group red spots together if their centroids are within 35 pixels (approximately 11 μm) of each other (see image processing methods, A1.1).

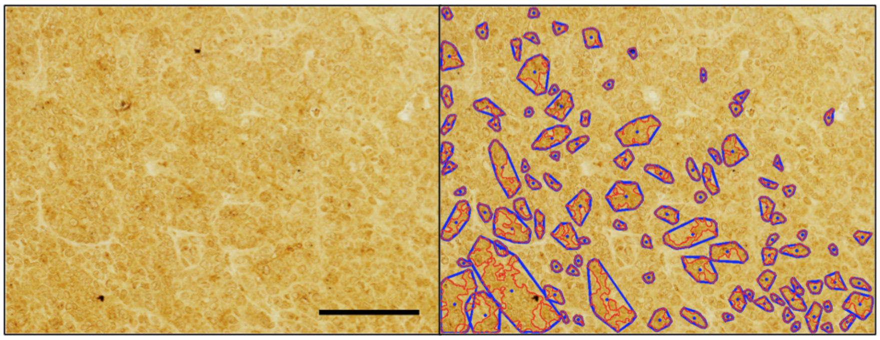

Appendix Figure S 4: Mock LEF1 contours (continued). Scalebars indicate 100μm, with each images' dimensions approximately $440 \times 330 \mu m^2$. The left column shows stained images. The right column shows stained images with red contour outlines drawn around each spot and blue convex hull outlines which group red spots together if their centroids are within 35 pixels (approximately 11 μm) of each other (see image processing methods, A1.1).

A1.7 SW480 Mock xenograft tumor β-catenin immunohistochemistry

The figures that follow are stains that were processed and whose data (area of each spot and distance to nearest neighbor) appear in the image analysis scatter plots in the main text.

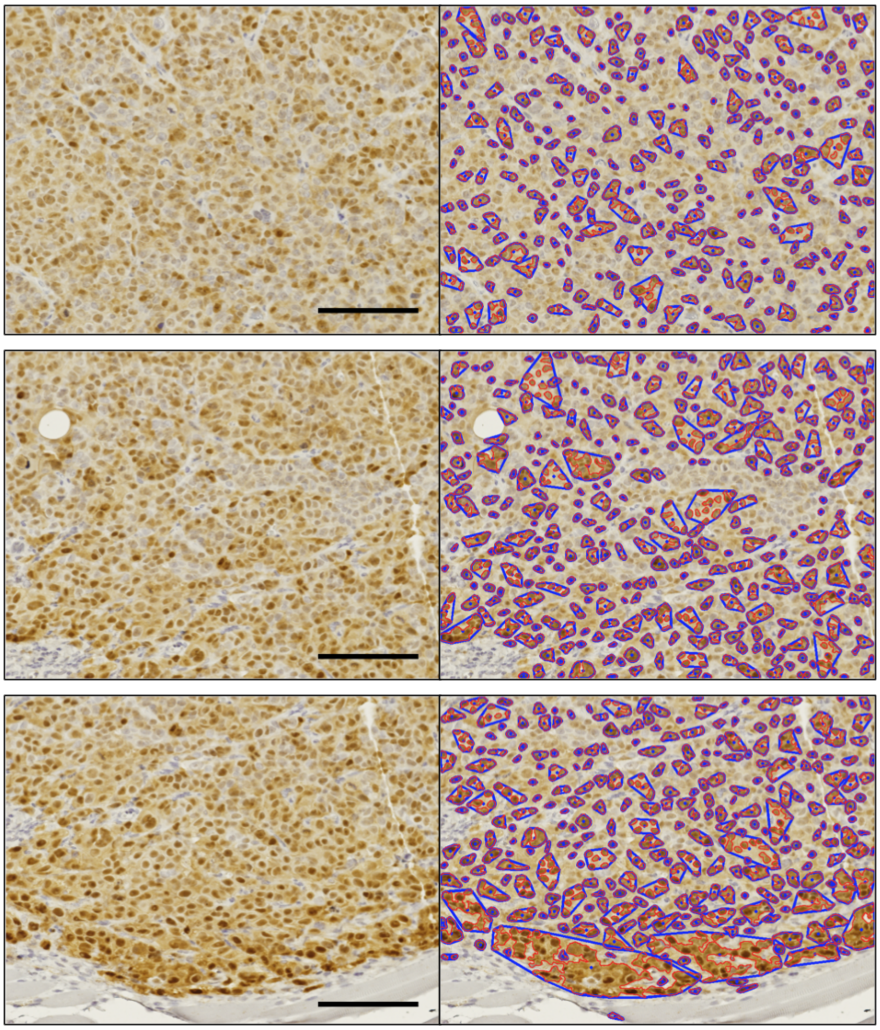

Appendix Figure S 5: Mock β-catenin contours. Scalebars indicate 100μm, with each images' dimensions approximately $440 \times 330\mu m^2$. The left column shows stained images. The right column shows stained images with red contour outlines drawn around each spot and blue convex hull outlines which group red spots together if their centroids are within 35 pixels (approximately 11 μm) of each other (see image processing methods, A1.1).

A1.8 SW480 dnLEF xenograft tumor phospho-PDH immunohistochemistry

The figures that follow are stains that were processed and whose data (area of each spot and distance to nearest neighbor) appear in the image analysis scatter plots in the main text (Figure 3D).

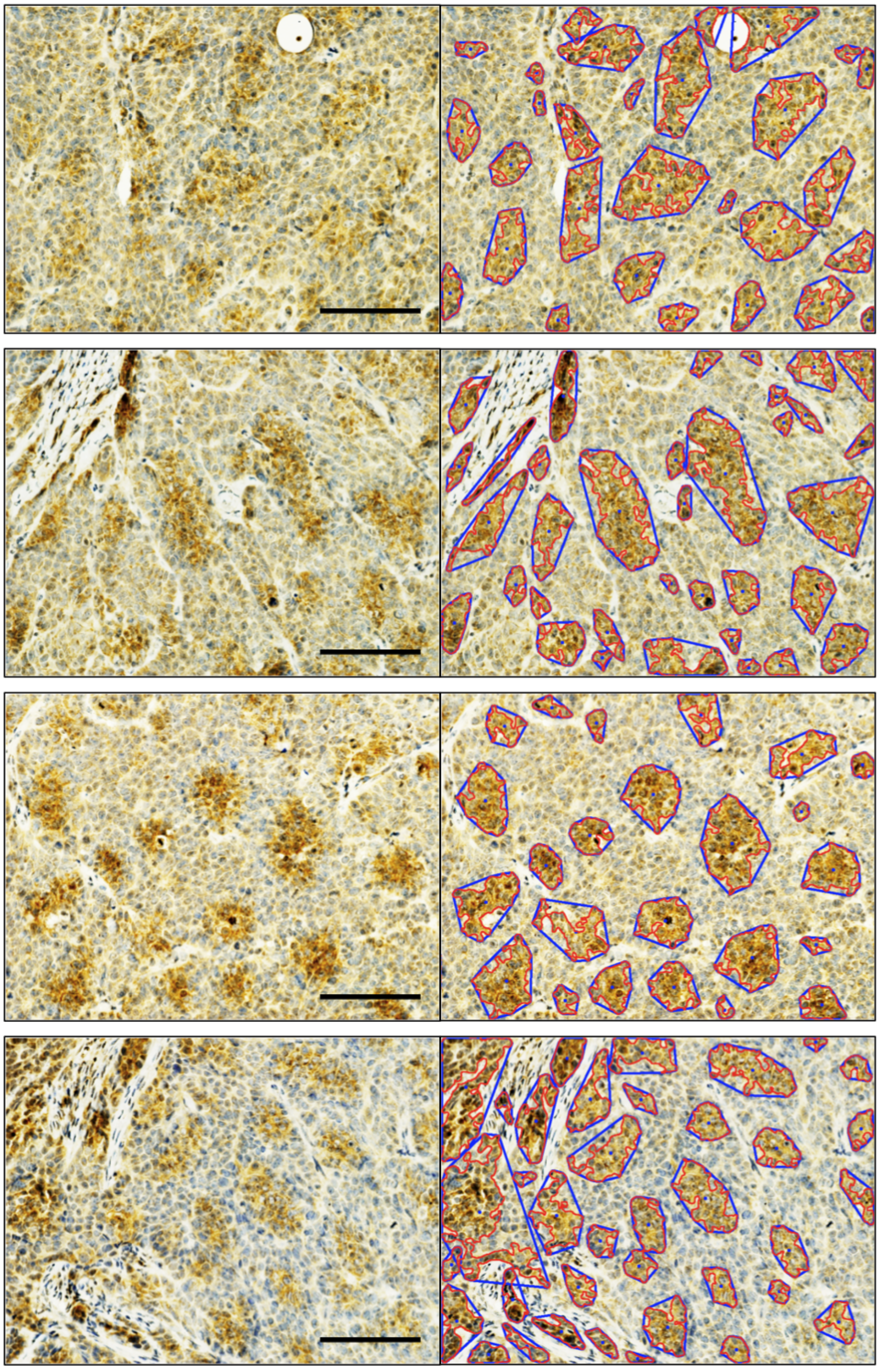

Appendix Figure S 6: dnLEF phospho-PDH contours. Scalebars indicate 100μm, with each images' dimensions approximately $440 \times 330 \mu m^2$. The left column shows stained images. The right column shows stained images with red contour outlines drawn around each spot and blue convex hull outlines which group red spots together if their centroids are within 35 pixels (approximately 11 μm) of each other (see image processing methods, A1.1).

A1.9 SW480 dnLEF xenograft tumor β-catenin immunohistochemistry

The figures that follow are stains that were processed and whose data (area of each spot and distance to nearest neighbor) appear in the image analysis scatter plots in the main text (Figure 3E).

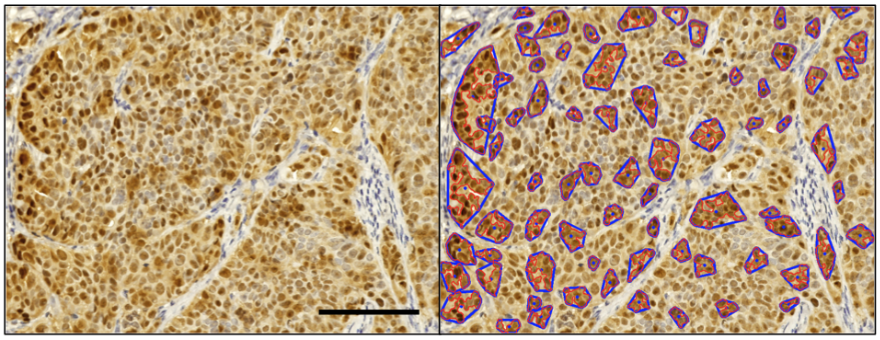

Appendix Figure S 7: dnLEF1 β-catenin contours. Scalebars indicate 100μm, with each images' dimensions approximately $440 \times 330 \mu m^2$. The left panel shows stained images. The right panel shows stained images with red contour outlines drawn around each spot and blue convex hull outlines that group red spots together if their centroids are within 35 pixels (approximately 11 μm) of each other (see image processing methods, A1.1). Spots highlighted here have high β-catenin expression in the cytoplasm and not just the nucleus.

A1.10 Heterogeneity in SW480 dnTCF xenograft tumors

Heterogeneity in metabolism and Wnt activity was also observed in dnTCF tumors. There is no counterstain for nuclei in these stains.

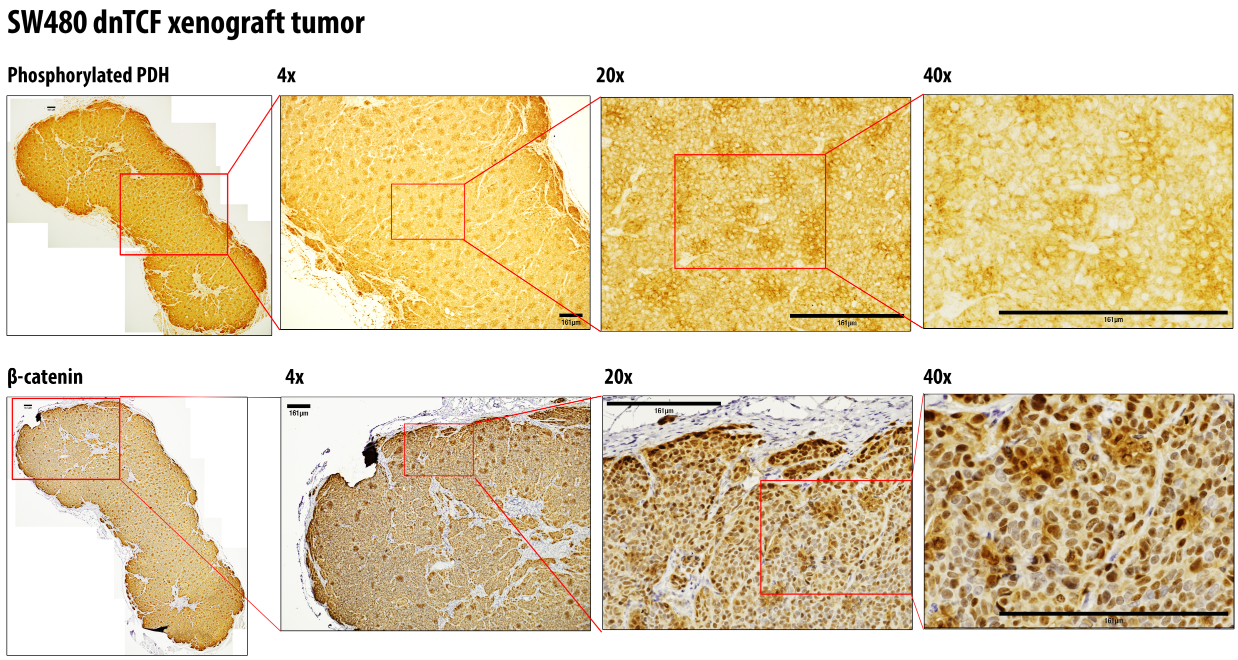

Appendix Figure S 8: Phospho-PDH and β-catenin immunohistochemical detection of expression in a dnTCF tumor. SW480 cells were lentivirally transduced to express dominant negative TCF1, which interferes with Wnt signaling activation of target genes. Transduced cells were injected subcutaneously into immunocompromised mice. Tumor sections were stained for phosphorylated PDH and beta-catenin. Scalebars are 161 μm.

A1.11 SW480 dnTCF phospho-PDH immunohistochemistry

The figures that follow are stains that were processed and whose data (area of each spot and distance to nearest neighbor) appear in the image analysis scatter plots in the main text (Figure 3D).

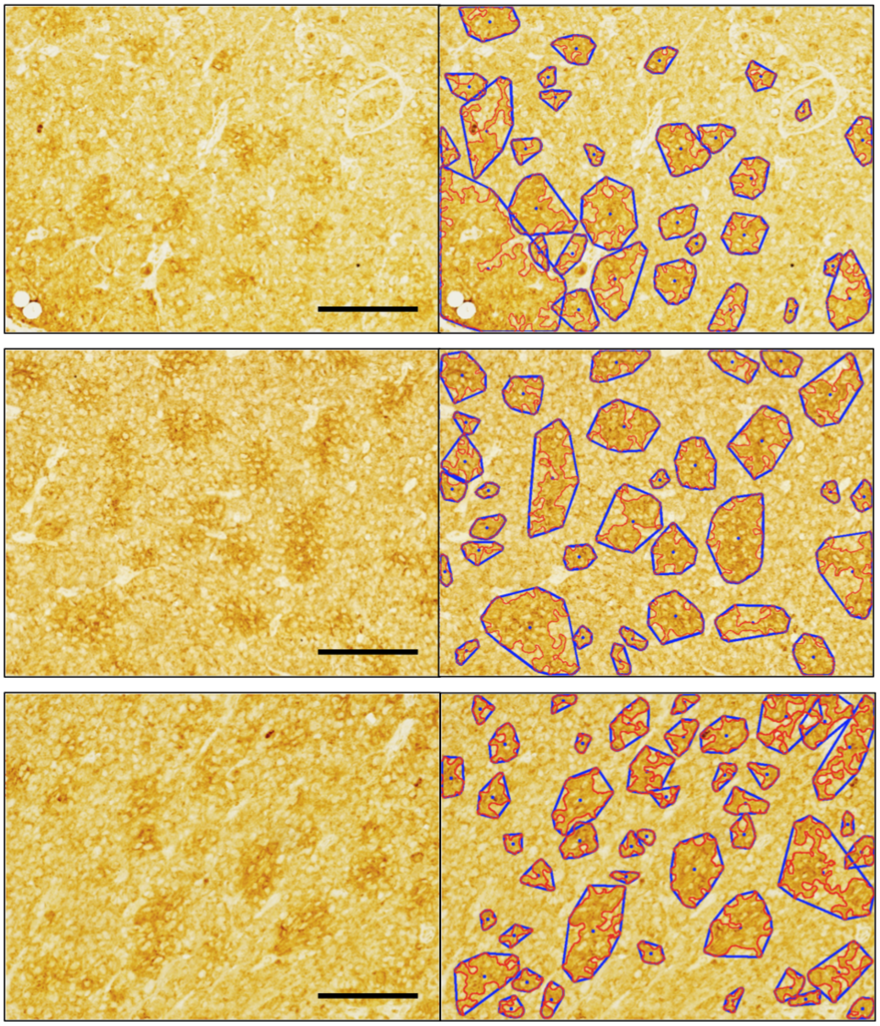

Appendix Figure S 9: dnTCF phospho-PDH contours. Scalebars indicate 100μm, with each images' dimensions approximately 440 $\times$ 330μm^2. The left column shows stained images. The right column shows stained images with red contour outlines drawn around each spot and blue convex hull outlines which group red spots together if their centroids are within 35 pixels (approximately 11 μm) of each other (see image processing methods, A1.1).

A1.12 SW480 dnTCF β-catenin immunohistochemistry

The figures that follow are stains that were processed and whose data (area of each spot and distance to nearest neighbor) appear in the image analysis scatter plots in the main text (Figure 3E).

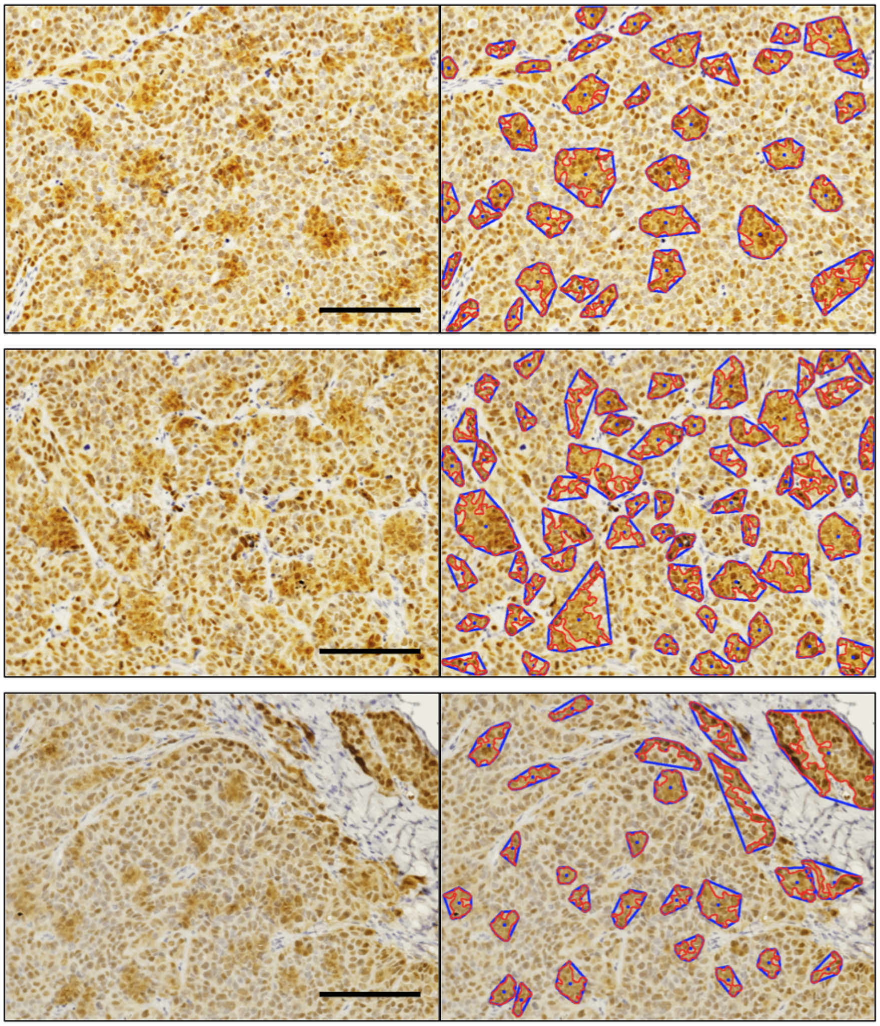

Appendix Figure S 10: dnTCF β-catenin contours. Scalebars indicate 100μm, with each images' dimensions approximately $440 \times 330\mu m^2$. The left column shows stained images. The right column shows stained images with red contour outlines drawn around each spot and blue convex hull outlines which group red spots together if their centroids are within 35 pixels (approximately 11 μm) of each other (see image processing methods, A1.1).

A1.13 Significance in differences between mock and dominant negative tumors in metabolic patterning

The p-values in the tables below were computed using the image processing data after combining spots whose centroids were more than 35 pixels apart (approximately 11 μm; see image processing description in previous section). Wilcoxon's rank-sum test was done using Matlab's ranksum.m function, which tests the statistical significance in the difference of the medians of two datasets, regardless of their distribution.

Appendix Table S 5: **p-values for area of spot**

	**mock pPDH**	**mock LEF1**	**mock β-catenin**	**dnLEF pPDH**	**dnTCF pPDH**	**dnLEF β-catenin**	**dnTCF β-catenin**
**mock pPDH**		3.91E-17	1.25E-68	6.87E-30	9.82E-21	0.0111	1.69E-20
**mock LEF1**			1.47E-43	2.40E-51	1.21E-41	5.03E-15	5.81E-45
**mock β-catenin**				1.93E-68	7.85E-59	1.96E-32	2.62E-64
**dnLEF pPDH**					0.0292	6.69E-16	6.79E-06
**dnTCF pPDH**						1.09E-10	0.1525
**dnLEF β-catenin**							9.54E-11

Appendix Table S 6: **p-values for distance to nearest neighbor**

	**mock pPDH**	**mock LEF1**	**mock β-catenin**	**dnLEF pPDH**	**dnTCF pPDH**	**dnLEF β-catenin**	**dnTCF β-catenin**
**mock pPDH**		8.15E-08	3.15E-100	3.31E-40	1.41E-23	1.40E-03	3.29E-21
**mock LEF1**			3.19E-84	7.74E-50	5.35E-33	1.01E-09	7.59E-32
**mock β-catenin**				7.81E-73	1.44E-62	2.33E-39	8.21E-67
**dnLEF pPDH**					2.70E-05	2.32E-19	2.48E-08
**dnTCF pPDH**						9.03E-10	0.24
**dnLEF β-catenin**							1.29E-07

A1.14 Description of image processing of simulation results

Simulations results were processed in the following way, after numerical solutions were obtained:

The P_g cell matrix was converted to 0's and 1's by setting matrix entries to 1 if greater than or equal to 0.99 and 0 otherwise. Areas and centroids were computed using Matlab's regionprops.m function. Distances to nearest neighbor (centroid-to-centroid) were then calculated. These distances are based on the simulation length scale, which we converted to a dimensional length scale using the following: 1 computational unit $= 37.5043$ μm. This length scale was chosen so that there was good agreement between experimental and simulation averages. Computations for the W matrix were similar, except the cutoff used was 8 (i.e., if $W \geq 8$, set the entry to 1, and 0 otherwise). The averages are plotted and labeled as simulation averages in the main text (P_g spots represent pPDH spots and W spots represent β-catenin spots).

A1.15 Quantification of IHC staining intensity and cells per spot in SW480 Mock and dnLEF pPDH staining

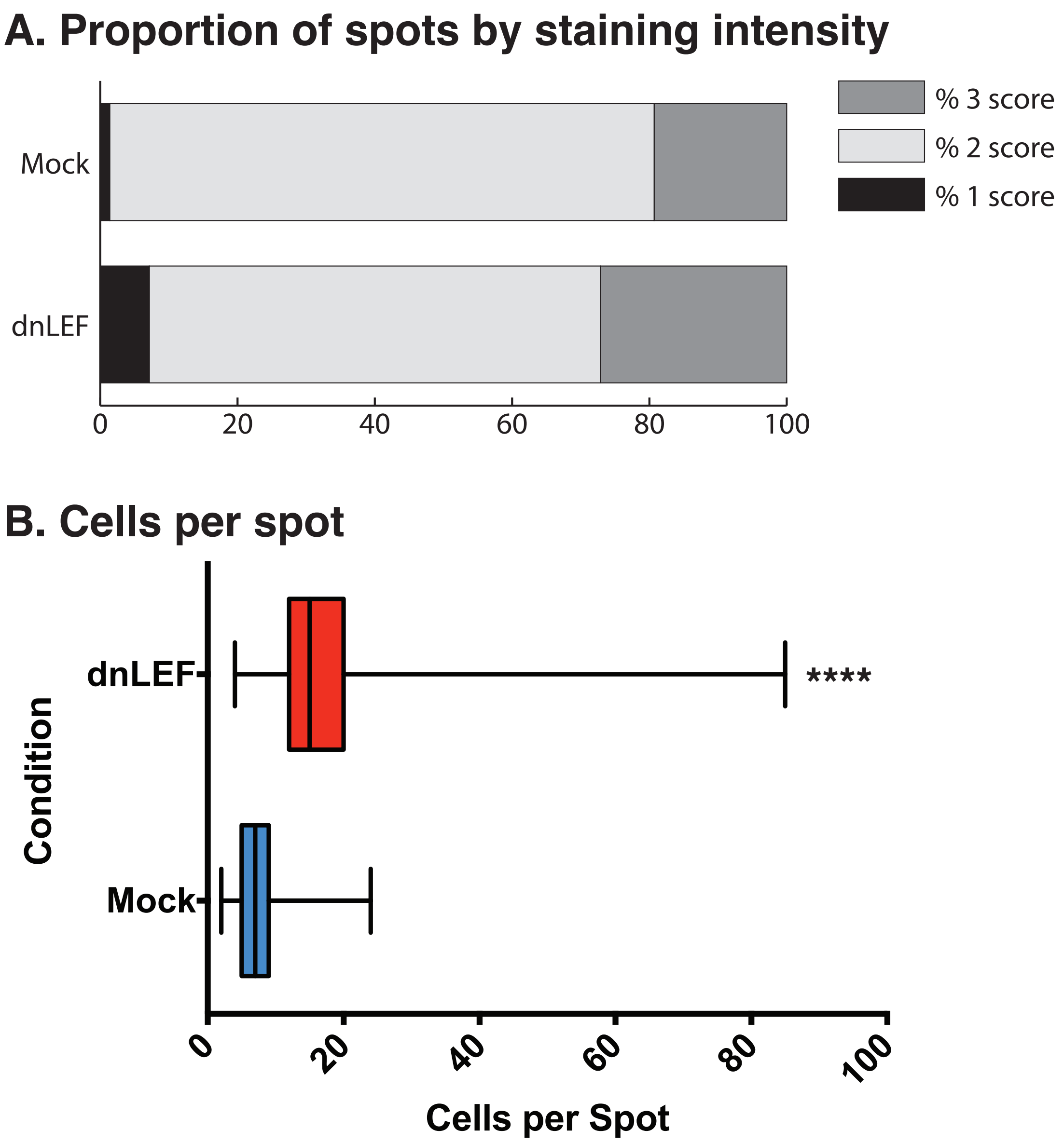

Appendix Figure S 11: Quantification of pPDH spots in SW480 Mock and dnLEF xenograft tumors for cells per tumor and intensity of staining. Three images each of SW480 Mock and dnLEF pPDH staining were provided to blinded researchers who were asked to identify the number of spots within each image and rate the staining intensity of each spot on a scale of 0 to 3, where 3 is the highest intensity. A. The proportion of spot scores for each tumor type. B. Cells per spot per tumor type.

A2 Nondimensionalization

The full set of dimensionalized equations are given by the following, where $\overline{P}$ is the characteristic cell population:

$$\frac{\partial P_o}{\partial t} = D_o \nabla^2 P_o + \frac{1}{\tau_o} N \left(1 - \frac{P_o + P_g + P_d}{\overline{P}}\right) P_o + \frac{1}{\tau_{go}} \chi_W(W) P_g \tag{1a}$$

$$- \frac{1}{\tau_{og}} \chi_W^*(W) \chi_N^*(N) P_o - \mu_o \chi_N(N) P_o \tag{1b}$$

$$\frac{\partial P_g}{\partial t} = D_g \nabla^2 P_g + \frac{1}{\tau_g} \frac{W}{\alpha_W + W} N \left(1 - \frac{P_o + P_g + P_d}{\overline{P}}\right) P_g - \frac{1}{\tau_{go}} \chi_W(W) P_g \tag{1c}$$

$$+ \frac{1}{\tau_{og}} \chi_W^*(W) \chi_N^*(N) P_o - \mu_g \chi_N(N) P_g \tag{1d}$$

$$\frac{\partial P_d}{\partial t} = D_d \nabla^2 P_d + \mu_o \chi_N(N) P_o + \mu_g \chi_N(N) P_g - \mu_d P_d \tag{1e}$$

$$\frac{\partial W}{\partial t} = D_W \nabla^2 W + \frac{1}{a + bW_I} \kappa_W N W^2 P_g + S_W(P_o + P_g) - \mu_W W \tag{1f}$$

$$\frac{\partial W_I}{\partial t} = D_{W_I} \nabla^2 W_I + \kappa_{W_I} N W^2 (P_o + P_g) - \mu_{W_I} W_I \tag{1g}$$

$$\frac{\partial N}{\partial t} = D_N \nabla^2 N - \nu_{NG} N P_g - \nu_{NO} N P_o - \mu_N N + N_s \tag{1h}$$

and

$$\chi_W(W) = \frac{1}{2}\left[1 - \tanh(\gamma_W(W - W^*))\right]$$
$$\chi_W^*(W) = \frac{1}{2}\left[1 + \tanh(\gamma_W(W - W^*))\right]$$
$$\chi_N(N) = \frac{1}{2}\left[1 - \tanh(\gamma_N(N - N_d))\right]$$
$$\chi_N^*(N) = \frac{1}{2}\left[1 + \tanh(\gamma_N(N - N_g^*))\right]$$

Introducing the nondimensional quantities below, where overbars indicate characteristic values, we have $P_o' = \frac{P_o}{\overline{P}}$, $P_g' = \frac{P_g}{\overline{P}}$, $P_d' = \frac{P_d}{\overline{P}}$, $W' = \frac{W}{\overline{W}}$, $W_I' = \frac{W_I}{\overline{W_I}}$, $N' = \frac{N}{\overline{N}}$.

We define nondimensionalized time as $t' = \frac{t}{T}$ and nondimensionalized length as $x' = \frac{x}{\ell}$, where $T = \frac{\tau_o}{\overline{N}}$ and $\ell = \sqrt{\frac{D_{W_I}\tau_o}{\overline{N}}}$.

We also define the following nondimensional parameters:

$$
\begin{aligned}
D'_o &= \frac{\tau_o D_o}{\overline{N}\ell^2} \\
\frac{1}{\tau'_{go}} &= \frac{\tau_o}{\overline{N}\tau_{go}} \\
\frac{1}{\tau'_{og}} &= \frac{\tau_o}{\overline{N}\tau_{og}} \\
\mu'_o &= \frac{\tau_o \mu_o}{\overline{N}} \\
D'_g &= \frac{\tau_o D_g}{\overline{N}\ell^2} \\
\frac{1}{\tau'_g} &= \frac{\tau_o}{\tau_g} \\
\mu'_g &= \frac{\tau_o \mu_g}{\overline{N}} \\
a' &= a \\
b' &= b\overline{W_I} \\
D'_W &= \frac{\tau_o D_W}{\overline{N}\ell^2} \\
\mu'_W &= \frac{\tau_o \mu_W}{\overline{N}} \\
\kappa'_W &= \tau_o \overline{P} \kappa_W \overline{W} \\
S'_W &= \frac{\tau_o S_W \overline{P}}{\overline{N}\,\overline{W}} \\
\kappa_{W_I} &= \frac{\overline{W_I}}{\tau_o \overline{P}\,\overline{W}^2} \\
\mu'_{W_I} &= \frac{\tau_o \mu_{W_I}}{\overline{N}} \\
W'^* &= \frac{W^*}{\overline{W}} \\
\gamma'_W &= \gamma_W \overline{W} \\
N'_d &= \frac{N_d}{\overline{N}} \\
N'^*_g &= \frac{N^*_g}{\overline{N}} \\
\gamma'_N &= \gamma_N \overline{N}
\end{aligned}
$$

We redefine the switch functions in the following way:

$$
\begin{aligned}
\chi'_W(W') &= \frac{1}{2}\left[1 - \tanh(\gamma'_W(W' - W'^*))\right] \\
\chi'^*_W(W') &= \frac{1}{2}\left[1 + \tanh(\gamma'_W(W' - W'^*))\right] \\
\chi'_N(N') &= \frac{1}{2}\left[1 - \tanh(\gamma'_N(N' - N'^*))\right] \\
\chi'^*_N(N') &= \frac{1}{2}\left[1 + \tanh(\gamma'_N(N' - N'^*_g))\right]
\end{aligned}
$$

Using these nondimensional quantities, and dropping the prime notation, the nondimensional equations are

$$\frac{\partial P_o}{\partial t} = D_o \nabla^2 P_o + N\left(1 - P_o - P_g - P_d\right) P_o + \frac{1}{\tau_{go}} \chi_W(W) P_g \tag{2a}$$

$$- \frac{1}{\tau_{og}} \chi^*_W(W) \chi^*_N(N) P_o - \mu_o \chi_N(N) P_o \tag{2b}$$

$$\frac{\partial P_g}{\partial t} = D_g \nabla^2 P_g + \frac{1}{\tau_g} \frac{W}{\alpha_W + W} N\left(1 - P_o - P_g - P_d\right) P_g - \frac{1}{\tau_{go}} \chi_W(W) P_g \tag{2c}$$

$$+ \frac{1}{\tau_{og}} \chi^*_W(W) \chi^*_N(N) P_o - \mu_g \chi_N(N) P_g \tag{2d}$$

$$\frac{\partial P_d}{\partial t} = D_d \nabla^2 P_d + \mu_o \chi_N(N) P_o + \mu_g \chi_N(N) P_g - \mu_d P_d \tag{2e}$$

$$\frac{\partial W}{\partial t} = D_W \nabla^2 W + \frac{1}{a + bW_I} \kappa_W N W^2 P_g + S_W(P_o + P_g) - \mu_W W \tag{2f}$$

$$\frac{\partial W_I}{\partial t} = \nabla^2 W_I + N W^2 \left(P_o + P_g\right) - \mu_{W_I} W_I \tag{2g}$$

$$\frac{\partial N}{\partial t} = D_N \nabla^2 N - \nu_{NG} N P_g - \nu_{NO} N P_o - \mu_N N + N_s \tag{2h}$$

A3 Wnt-PDK-Lactate-HIF1α Cross-Feeding Model

The model presented in this paper was developed to have minimal complexity. Because other signaling pathways and growth or inhibition factors are known to be involved in cancer growth and metabolism, we considered an extension to the Wnt signaling model to include these additional effects.

A3.1 Augmenting the Wnt Signaling Model

We added more detail to the Wnt signaling model by including equations for PDK, lactate, and HIF. The equations for P_o, P_g, P_d, W, W_I, and N (Equations 3b through 3k) are identical to those in the Wnt signaling model in the main text, except that the metabolic switch between OXPHOS and glycolysis is regulated by PDK activity rather than Wnt levels. If PDK is high, then the cells are more likely to switch to glycolysis, and if PDK is low, the cells are more likely to switch to OXPHOS. This larger model was built with the assumptions that Wnt and the hypoxia transcription factor HIF1α promote PDK expression and activity (**?**), (**?**), (**?**), PDK activity promotes lactate production through upregulation of glycolysis (**?**), and lactate increases HIF production (**?**).

The equation for P, PDK activity, is given by equation (3h). The first term on the right of the equality is random motion; the second term represents nonlinear upregulation by Wnt, since Wnt signaling upregulates PDK (**?**); the third term represents upregulation by HIF1α (**?**), (**?**); the fourth term stands for upregulation through the cells, with sufficient nutrient; and the last term is a decay term.

Equation (3i) is the equation for lactate, which is assumed to diffuse long-range; is upregulated nonlinearly by PDK through the P_g cells, since PDK drives glycolysis (**?**); and can decay and be uptaken by P_o cells, a form of metabolic symbiosis or cross feeding.

The dynamics for HIF are given by Equation (3j). HIF is assumed to diffuse, to be upregulated nonlinearly by lactate, and to decay. The nonlinear upregulation by lactate comes from the assumption that lactate stabilizes HIF (**?**). The last two terms in the HIF equation are production terms. There is a small rate (d_H) at which HIF is produced everywhere; and there is a large rate (d_{Hni}) at which HIF is produced when nutrient is low, which represents the stabilization of HIF in hypoxic environments.

$$\frac{\partial P_o}{\partial t} = \underbrace{D_o \nabla^2 P_o}_{\text{random motion}} + \underbrace{\frac{1}{\tau_o}\left(N + \frac{L}{L_s}\right)(1 - P_o - P_g - P_d)\, P_o}_{\text{proliferation}} - \underbrace{\mu_o \chi_N(N) P_o}_{\text{death}} \tag{3a}$$

$$+ \underbrace{\frac{1}{\tau_{go}} \chi_P(P) P_g}_{\text{switch to OXPHOS}} - \underbrace{\frac{1}{\tau_{og}} \chi_P^*(P) \chi_N^*(N) P_o}_{\text{switch from OXPHOS}} \tag{3b}$$

$$\frac{\partial P_g}{\partial t} = \underbrace{D_g \nabla^2 P_g}_{\text{random motion}} + \underbrace{\frac{1}{\tau_g} \frac{W}{\alpha_W + W} N (1 - P_o - P_g - P_d)\, P_g}_{\text{proliferation}} - \underbrace{\mu_g \chi_N(N) P_g}_{\text{death}} \tag{3c}$$

$$- \underbrace{\frac{1}{\tau_{go}} \chi_P(P) P_g}_{\text{switch from glycolysis}} + \underbrace{\frac{1}{\tau_{og}} \chi_P^*(P) \chi_N^*(N) P_o}_{\text{switch to glycolysis}} \tag{3d}$$

$$\frac{\partial P_d}{\partial t} = \underbrace{D_d \nabla^2 P_d}_{\text{random motion}} + \underbrace{\mu_o \chi_N(N) P_o}_{\text{dead } P_o \text{ cells}} + \underbrace{\mu_g \chi_N(N) P_g}_{\text{dead } P_g \text{ cells}} - \underbrace{\mu_d P_d}_{\text{decay}} \tag{3e}$$

$$\frac{\partial W}{\partial t} = \underbrace{D_W \nabla^2 W}_{\text{random motion}} + \underbrace{\frac{1}{a + bW_I}}_{\text{inhibition}} \underbrace{\kappa_W N W^2 P_g}_{\text{upregulation}} + \underbrace{S_W (P_o + P_g)}_{\text{upregulation}} - \underbrace{\mu_W W}_{\text{downregulation}} \tag{3f}$$

$$\frac{\partial W_I}{\partial t} = \underbrace{D_{W_I} \nabla^2 W_I}_{\text{random motion}} + \underbrace{\kappa_{W_I} N W^2 (P_o + P_g)}_{\text{upregulation}} - \underbrace{\mu_{W_I} W_I}_{\text{downregulation}} \tag{3g}$$

$$\frac{\partial P}{\partial t} = \underbrace{D_P \nabla^2 P}_{\text{random motion}} + \underbrace{\nu_{PW} \frac{W^2}{\alpha_{PW} + W} N}_{\text{upregulation}} + \underbrace{\nu_{PH} \frac{H}{\alpha_{PH} + H} N}_{\text{upregulation}} + \underbrace{\nu_P N (P_o + P_g)}_{\text{upregulation}} - \underbrace{\mu_P P}_{\text{downregulation}} \tag{3h}$$

$$\frac{\partial L}{\partial t} = \underbrace{D_L \nabla^2 L}_{\text{random motion}} + \underbrace{\nu_{LP} P_g \frac{P^2}{\alpha_P + P} N}_{\text{production}} - \underbrace{\mu_L L}_{\text{decay}} - \underbrace{\nu_{LO} L P_o}_{\text{uptake by } P_o \text{ cells}} \tag{3i}$$

$$\frac{\partial H}{\partial t} = \underbrace{D_H \nabla^2 H}_{\text{random motion}} + \underbrace{\nu_{HL} \frac{L^2}{\alpha_L + L}}_{\text{lactate-induced production}} - \underbrace{\mu_H H}_{\text{decay}} + \underbrace{d_H}_{\text{bulk production}} + \underbrace{d_{HNi} (1 - N)}_{\text{hypoxia-induced production}} \tag{3j}$$

$$\frac{\partial N}{\partial t} = \underbrace{D_N \nabla^2 N}_{\text{random motion}} - \underbrace{\nu_{NG} N P_g}_{\text{uptake by } P_g \text{ cells}} - \underbrace{\nu_{NO} N P_o}_{\text{uptake by } P_o \text{ cells}} - \underbrace{\mu_N N}_{\text{decay}} + \underbrace{N_s}_{\text{bulk source}} \tag{3k}$$

A3.2 Results

The parameters used in the results shown here are given by Table 7. The system evolves over time and its final output at time 50 is shown in Figures 12-13. The numerical results are similar to those in Figure 2 and 3, with a striking spotted pattern in glycolysis and OXPHOS. Near the boundary, there is a very high level of glycolysis, with localized spots of high glycolysis surrounded by relatively lower levels. We also see this spotted pattern in PDK activity, lactate, and HIF. When the mock and dnLEF tumors were stained for HIF, we saw a spotted pattern in both, with the dnLEF tumor exhibiting larger and fewer spots (see Supplemental Figure 14).

Appendix Table S 7: **Wnt-PDK-Lactate-HIF1α Model Parameters**

**Parameter**	**Description**	**Mock value**	**dnLEF value**
D_o	Diffusion coefficient of oxidative cells	0.01	0.01
D_g	Diffusion coefficient of glycolytic cells	0.01	0.01
D_W	Diffusion coefficient of Wnt	0.01	0.035
D_{W_I}	Diffusion coefficient of Wnt inhibitor	1	1.5
D_P	Diffusion coefficient of PDK activity	0.01	0.01
D_L	Diffusion coefficient of lactate	100	100
D_H	Diffusion coefficient of HIF	0.01	0.01
D_N	Diffusion coefficient of nutrient	100	100
τ_o	Oxidative cell proliferation time	1	1
τ_g	Glycolytic cell proliferation time	1	1
τ_{og}	Switch time from OXPHOS to glycolysis	1/24	1/24
τ_{go}	Switch time from glycolysis to OXPHOS	1	1
α_W	Constant for Michaelis-Menten dynamics	1	1
κ_W	Rate of nonlinear Wnt production	5	5
κ_{W_I}	Rate of Wnt inhibitor production	1	1
μ_o	Decay rate of P_o cells	1	1
μ_g	Decay rate of P_g cells	1	1
μ_d	Decay rate of P_d cells	1	1
μ_W	Decay rate of Wnt	3	3
μ_{W_I}	Decay rate of Wnt inhibitor	5	5
μ_P	Decay rate of PDK activity	1	1
μ_L	Decay rate of lactate	1000	1000
μ_H	Decay rate of HIF	1	1
μ_N	Decay rate of nutrient	1	1
ν_{PW}	Rate of PDK upregulation through Wnt	2	2
ν_{PH}	Rate of PDK upregulation through HIF	0.5	0.5
ν_{LP}	Rate of lactate upregulation thrugh PDK activity	3000	3000
ν_{HL}	Rate of HIF stabilization through lactate	3	3
ν_P	PDK activity upregulation through cells	1	1
ν_{LO}	Rate of lactate uptake by P_o cells	10^5	10^5
d_H	Constitutive HIF production	0.5	0.5
d_{HNi}	Rate of HIF stabilization due to low nutrient	5	5
S_W	Rate of Wnt production through cells	7.5	5.5
a	Constant of inhibition	10^{-8}	10^{-8}
b	Constant of inhibition by W_I	1	1
γ_P	Sensitivity level of PDK switch functions	1	1
γ_N	Sensitivity level of nutrient switch function	100	100
ν_{NG}	Uptake of nutrient by P_g cells	100	100
ν_{NO}	Uptake of nutrient by P_o cells	100	100
N_s	Parameter for nutrient source	30	30
L_s	Parameter for characteristic value of L	1	1
P^*	PDK activity level at which 50% of cells switch metabolism	3	3
N_d	Nutrient level below which cells will die	0.07	0.07
N_g^*	Nutrient level below which P_o cells cannot switch to glycolysis	0.1	0.1
α_N	Value of scaling function when $\int P_g = 0$	0.025	0.025
S_x	Horizontal length of spatial domain	12	12
S_y	Vertical length of spatial domain	12	12

Model parameters for mock and dnLEF/dnTCF simulations

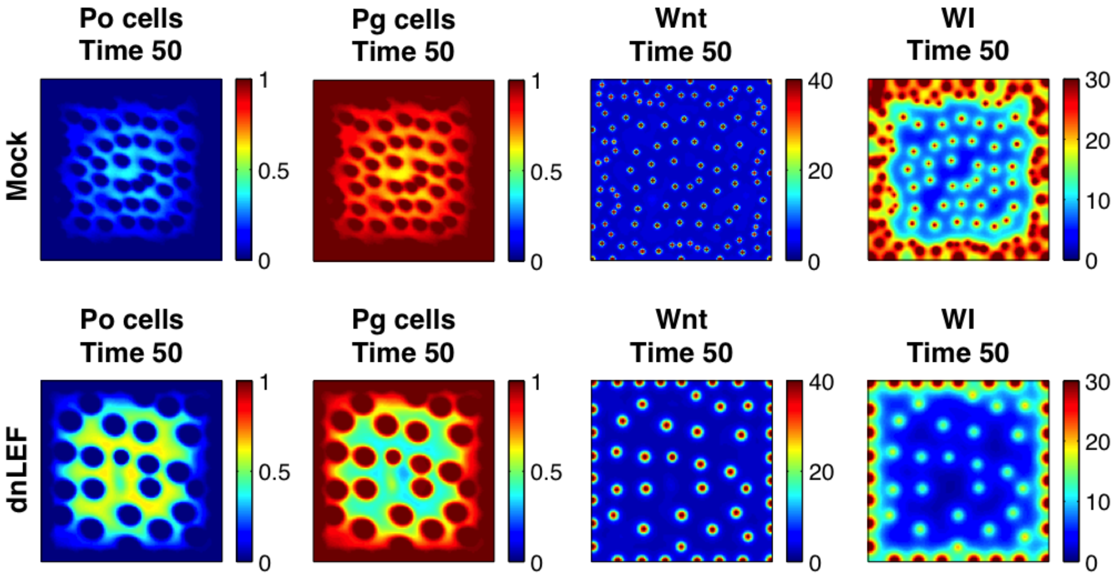

Appendix Figure S 12: Numerical results for mock and dnLEF P_o, P_g, W, and W_I in Wnt-PDK-lactate-HIF1α model.

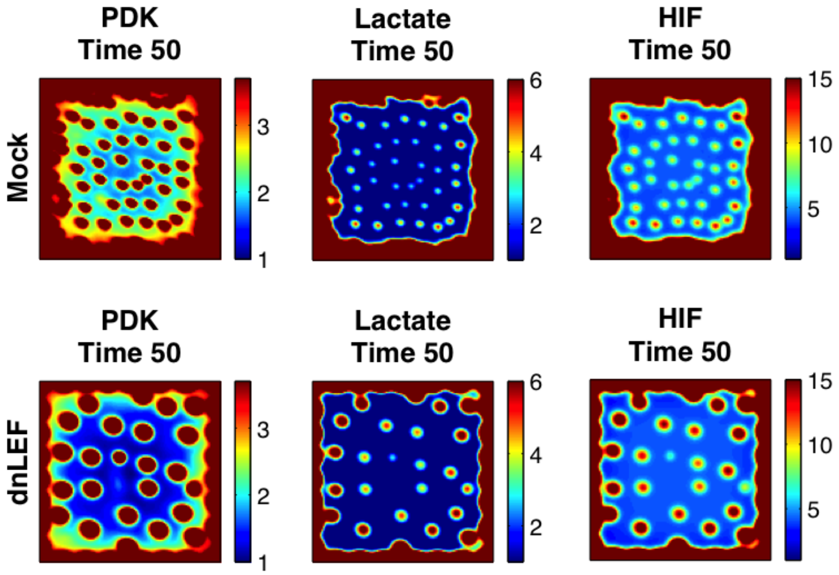

Appendix Figure S 13: Numerical results for mock and dnLEF PDK, lactate, and HIF in Wnt-PDK-lactate-HIF1α model.

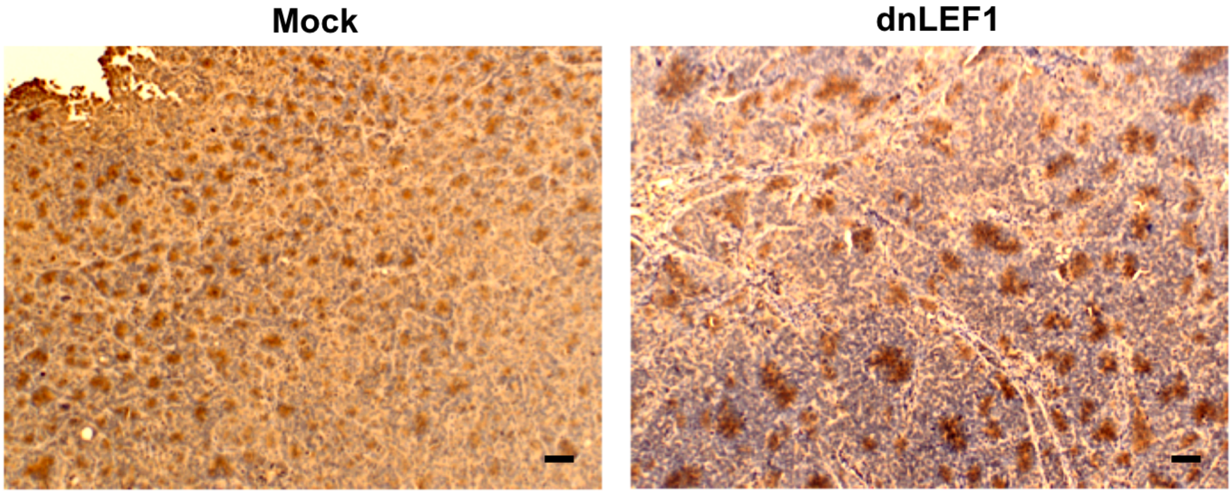

Appendix Figure S 14: Stains for HIF1α in mock and dnLEF tumors. Scalebars indicate 100μm

A3.3 Discussion

The results from this more detailed model give us good qualitative agreement with our IHC data when we compare the numerical results for PDK activity with our phospho-PDH stains. We see the larger, fewer spots and lighter background in the dominant negative LEF PDK results in comparison to mock. It was not possible to stain for lactate, but experimental data show that the cells and HIF1α follow the same spotted pattern, and the same change in the pattern from mock to dnLEF/dnTCF, and we therefore conclude that this model is sufficient to recapitulate qualitatively our experimental observations.

Importantly, this more detailed model gives similar qualitative results to the simpler model presented in the main text when the experimental results are compared to numerical results for PDK activity. Since PDK drove switching in the cells, we saw patterns in metabolism as well. The simpler model retains the most important elements of the larger model while producing similar results. In the Wnt signaling model discussed in the main text, the positive feedback between Wnt and PDK (high PDK implies more P_g cells; higher levels of P_g cells imply more Wnt activity; more Wnt activity means increased PDK) has been distilled so that Wnt activity level is the effective switch rather than PDK. Because PDK was directing the switch in metabolism, we can use P_g and P_o as the effective patterned state to compare to the xenograft stains. Thus, the equations for PDK activity, HIF1α concentration, and lactate concentration can be removed from the system, with Wnt activity driving the switch rather than PDK, so that the remaining equations contain only most important elements of the model.

A3.4 Numerical method

Numerical simulations were performed in MATLAB, using a forward difference method for each time derivative. P_o, P_g, W, W_I, P, L, and H equations were solved implicitly in centered diffusion terms. The nutrient equation was solved implicitly in uptake, decay, and centered diffusion terms. No-flux boundary conditions were used for all fields except for the nutrient boundary which used a Dirichlet boundary condition. The initial condition for the P_g cell population was a random distribution near the boundary of the domain. W and W_I initial conditions were a random distribution in the same locations where initial P_g cells were located. Initial N was 1 everywhere in the domain; results did not change qualitatively if N was solved as a quasi-steady state equation. Initial conditions for P_o, P_d, P, L, and H terms were 0.

A4 Nutrient diffusion simulation

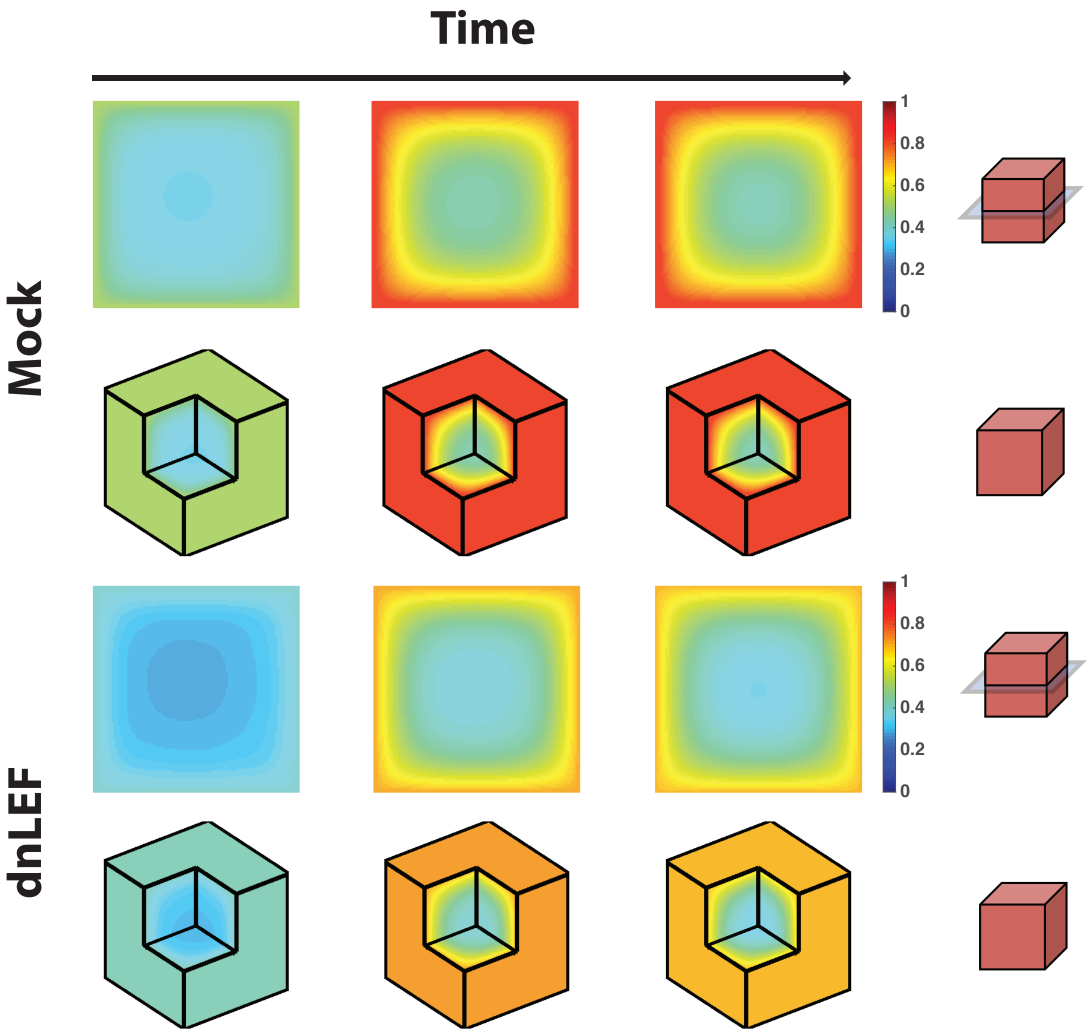

Appendix Figure S 15: Nutrient diffusion simulation for Wnt signaling model. Nutrients diffuse into the system form all boundaries. The rate of diffusion and total concentration of nutrient is influenced by Wnt signaling and metabolic program switching.

A5 Parameter exploration

This table lists the set of parameters we investigated in the Wnt signaling model in order to test our model for robustness. The parameters that are missing from this table were explored in the main part of the paper, so their effects are already evaluated and are not included here. We varied each parameter one by one, varying between the minimum and maximum values listed in the table, and kept all other values the same as the mock values in the table of parameter values in the main text, to ensure that a pattern would emerge that was qualitatively consistent with our mock stains. Next, we made the same changes from mock to dnLEF as presented in the paper (increased the diffusion coefficients for W and W_I and decreased S_W) to see if we still obtained qualitatively similar answers as the dnLEF/dnTCF experiments. In this way we determine whether our model is robust to a large range of values, so that the results we obtain from the parameters presented in the main part of this paper can be considered characteristic of our system.

Appendix Table S 8: **Parameter sensitivity study**

**Parameter**	**Mock value**	**dnLEF value**	**Minimum**	**Maximum**	**Comment**
κ_W	5	5	3	50	$\kappa_W = 2$: no pattern in dnLEF
κ_{W_I}	1	1	0.1	1.25	$\kappa_{W_I} = 1.5$: no pattern in dnLEF
a	10^{-8}	10^{-8}	10^{-16}	0.75	$a = 1$: no pattern in dnLEF
b	1	1	0.1	1.75	$b = 2$: no pattern in dnLEF
τ_{og}	1/24	1/24	1/85	1/20	$\tau_{og} = 1/18$: no pattern in dnLEF; $\tau_{og} = 1/90$: almost all P_g; not enough spots in mock
μ_o	1	1	0	40	$\mu_o = 50$: no pattern in dnLEF
μ_g	1	1	0	100	Did not try larger than 100
μ_d	1	1	0	100	Did not try larger values
α_W	1	1	10^{-8}	1.5	Numerical error if $\alpha_W = 0$; no pattern in dnLEF if $\alpha_W = 2$
μ_W	2	2	2	2	$\mu_W = 1$, dnLEF: pattern in Wnt but none in P_g (100% P_g); $\mu_W = 1.5$, dnLEF: slight pattern in P_g (almost 100% P_g); $\mu_W = 1.75$, mock: slight pattern in P_g (almost 100% P_g, but fewer spots than in dnLEF); $\mu_W = 2.25$, dnLEF: all terms $\to 0$
μ_{WI}	3	3	3	20	$\mu_{WI} = 2.75$, dnLEF: no pattern; $\mu_{WI} = 20$, mock: almost 100% P_g, 3 "spots"
ν_{NG}	10	10	0	25	$\nu_{NG} = 30$: no pattern in dnLEF
ν_{NO}	10	10	0	20	$\nu_{NO} = 25$: no pattern in dnLEF
μ_N	0.1	0.1	0	1	$\mu_N = 1.5$: no pattern in dnLEF
N_s	2	2	0	10	$N_s = 10$: nutrient essentially constant throughout domain in dnLEF and mock
L_s	65.9	57.7	1/100	100	Small values of L_s result in the pattern forming more quickly

Robustness to parameter changes: The minimum and maximum values listed in this table indicate the range of tested values for which we still see the same characteristic phenotype changes in our model. This means that from mock to dnLEF, we observe fewer, larger spots farther apart from each other.

A6 Diffusive stability analysis

We chose our parameters first by looking only at a modified version of the two equation Gierer-Meinhardt activator-inhibitor system, given by

$$\frac{\partial a}{\partial t} = D_a \nabla^2 a + \kappa_a \frac{a^2}{h} - \mu_a a + \rho_a$$
$$\frac{\partial h}{\partial t} = D_h \nabla^2 h + \kappa_h a^2 - \mu_h h + \rho_h$$

where a is the activator that creates its own inhibitor h (modified so that the rates for nonlinear production of a and h can be different). Classic diffusive instability analysis was done on this smaller system to find parameters that expect to give us patterns. After finding and fixing suitable parameters for these equations, we performed diffusive stability analysis on the larger system by solving the equations without diffusion using MATLAB's ode45 solver and 100 time steps, fixing other values and varying two parameters at a time. If a steady state was not found, then that set of parameters was assumed to give us oscillations and so the linear stability analysis could not be performed with those parameters. If a steady state was found, we linearized around that steady state and we determined whether it was stable or not by looking at the real part of each eigenvalue. If all real parts were negative, we include diffusion and if any these new eigenvalues had a positive real part, then that set of parameters was expected to give us a pattern.

In the figures that follow, areas that are white are associated with parameters where no patterns are expected to form. Areas that are neither white nor blue show the eigenvalue and wavenumber associated with the pattern that is predicted to emerge. Any area that is blue is a region in which the solution of the ordinary differential equation (ODE) versions of the equations is oscillatory rather than steady, hence the equations could not be linearized about a steady state and so the analysis could not be performed. That is not to say, however, that patterns could not form with those parameter values.

We plot eigenvalues, which quantify the instability of an initial perturbation, and wavenumbers, which predict the dominant number of spikes or peaks in the entire system. Note that each wavenumber graph is plotted with a fixed colorscale between white (0) and black (12); wavenumbers above 12 were not tested. If a set of parameters has a nonzero eigenvalue, it is expected that a perturbation grows in time, hence a pattern should form in early times. The higher the eigenvalue, the higher the instability and hence more likelihood for a pattern to emerge and remain over the long term. Hence, a nonzero eigenvalue that is very small may yield a pattern only in very early times in the simulation. Moreover, a nonzero eigenvalue and wavenumber may mean a pattern in Wnt and inhibitor, but this may not necessarily translate to a pattern in the cells.

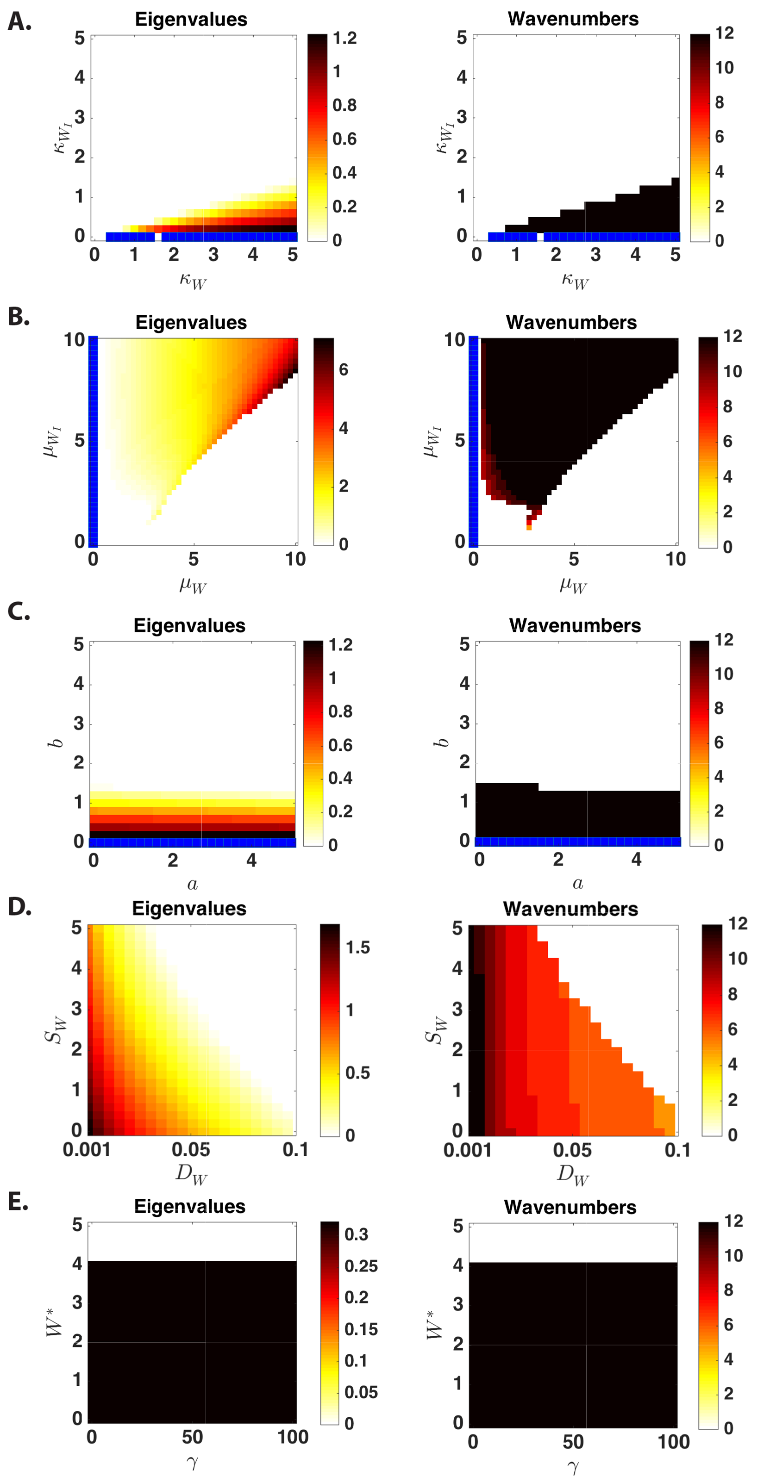

Appendix Figure S 16: Diffusive instability analysis A. Diffusive instability analysis varying κ_W and κ_{W_I}. B. Analysis varying μ_W and μ_{W_I}. C. Analysis varying a and b. D. Analysis varying D_W and S_W. E. Analysis varying γ and W^*.

A7 Simulation of in vitro tumor growth with DCA and XAV939 treatment

A7.1 Boundary Conditions

N is set to Dirichlet constant 0.25. P_g, P_o, P_d, W, W_I, L is Neumann.

A7.2 Equations

$$\frac{\partial P_o}{\partial t} = \underbrace{D_o \nabla^2 P_o}_{\text{random motion}} + \underbrace{\frac{1}{\tau_o}\left(N + \frac{L}{L_s}\right)(1 - P_o - P_g - P_d)P_o}_{\text{proliferation}} - \underbrace{\mu_o \chi_{NL}(N, L) P_o}_{\text{death}} \tag{4}$$

$$+ \underbrace{\frac{1}{\tau_{go}} \chi_W(W) P_g}_{\text{switch to OXPHOS}} - \underbrace{\frac{1}{\tau_{og}} \chi^*_W(W) \chi^*_N(N) P_o}_{\text{switch from OXPHOS}} \tag{5}$$

$$\frac{\partial P_g}{\partial t} = \underbrace{D_g \nabla^2 P_g}_{\text{random motion}} + \underbrace{\frac{1}{\tau_g} \frac{W}{\alpha_W + W} N(1 - P_o - P_g - P_d)P_g}_{\text{proliferation}} - \underbrace{\mu_g \chi_N(N) P_g}_{\text{death}} \tag{6}$$

$$- \underbrace{\frac{1}{\tau_{go}} \chi_W(W) P_g}_{\text{switch from glycolysis}} + \underbrace{\frac{1}{\tau_{og}} \chi^*_W(W) \chi^*_N(N) P_o}_{\text{switch to glycolysis}} \tag{7}$$

$$\frac{\partial P_d}{\partial t} = \underbrace{D_d \nabla^2 P_d}_{\text{random motion}} + \underbrace{\mu_o \chi_{NL}(N, L) P_o}_{\text{dead } P_o \text{ cells}} + \underbrace{\mu_g \chi_N(N) P_g}_{\text{dead } P_g \text{ cells}} - \underbrace{\mu_d P_d}_{\text{decay}} \tag{8}$$

$$\frac{\partial W}{\partial t} = \underbrace{D_W \nabla^2 W}_{\text{random motion}} + \underbrace{\frac{1}{a + bW_I}}_{\text{inhibition}} \underbrace{\kappa_W N W^2 P_g}_{\text{upregulation}} + \underbrace{S_W(P_o + P_g)}_{\text{upregulation}} - \underbrace{\mu_W W}_{\text{downregulation}} \tag{9}$$

$$\frac{\partial W_I}{\partial t} = \underbrace{D_{W_I} \nabla^2 W_I}_{\text{random motion}} + \underbrace{\kappa_{W_I} N W^2 (P_o + P_g)}_{\text{upregulation}} - \underbrace{\mu_{W_I} W_I}_{\text{downregulation}} \tag{10}$$

$$\frac{\partial N}{\partial t} = \underbrace{D_N \nabla^2 N}_{\text{random motion}} - \underbrace{\nu_{NG} N P_g}_{\text{uptake by } P_g \text{ cells}} - \underbrace{\nu_{NO} N P_o}_{\text{uptake by } P_o \text{ cells}} - \underbrace{\mu_N N}_{\text{decay}} \tag{11}$$

$$\frac{\partial L}{\partial t} = \underbrace{D_L \nabla^2 L}_{\text{random motion}} - \underbrace{\nu_{LO} L P_o}_{\text{uptake by } P_o \text{ cells}} + \underbrace{N_s P_g}_{\text{bulk source by } P_g} \tag{12}$$

(13)

Bulk source term:

$$N_s = \overline{N_s}$$

Switch functions:

$$\chi_{_W}(W) = \frac{1}{2}\left(1 - \tanh(\gamma_{_W}(W - W^*))\right)$$

$$\chi_{_W}^*(W) = \frac{1}{2}\left(1 + \tanh(\gamma_{_W}(W - W^*))\right)$$

$$\chi_{_{NL}}(N, L) = \frac{1}{2}\left(1 - \tanh\left(\gamma_{_N}\left((rN + (1-r)\frac{\gamma_{_L} L}{1 + \gamma_{_L} L}) - N^*\right)\ \right)\ \right)$$

$$\chi_{_N}^*(N) = \frac{1}{2}\left(1 + \tanh(\gamma_{_N}(N - N_g^*))\right)$$

A7.3 Parameter values for in vitro model

Parameter	Description	Mock value	dnLEF value
D_o	Diffusion coefficient of oxidative cells	0.01	0.01
D_g	Diffusion coefficient of glycolytic cells	0.01	0.01
D_d	Diffusion coefficient of dead cells	0	0
D_W	Diffusion coefficient of Wnt	0.004	0.008
D_{W_I}	Diffusion coefficient of Wnt inhibitor	1	1.5
D_N	Diffusion coefficient of Nutrient	100	100
D_L	Diffusion coefficient of L	100	100
τ_o	Oxidative cell proliferation time	1	1
τ_g	Glycolytic cell proliferation time	1	1
τ_{og}	Switch time from OXPHOS to glycolysis	$\frac{1}{24}$	$\frac{1}{24}$
τ_{go}	Switch time from glycolysis to OXPHOS	1	1
α_W	Constant for Michaelis-Menten dynamics	1	1
κ_W	Rate of nonlinear Wnt production	5	5
κ_{W_I}	Rate of nonlinear Wnt inhibitor production	1	1
μ_o	Decay rate of P_o cells	1	1
μ_g	Decay rate of P_g cells	1	1
μ_d	Decay rate of P_d cells	1	1
μ_W	Decay rate of Wnt	2	2
μ_{W_I}	Decay rate of Wnt inhibitor	3	3
μ_{W_I}	Decay rate of nutrient	0.1	0.1
S_W	Rate of Wnt production through cells	7.5	6.5
a	Constant of inhibition	10^{-8}	10^{-8}
b	Constant of inhibition by W_I	1	1
γ_W	Sensitivity level of Wnt switch functions	1	1
γ_N	Sensitivity level of nutrient switch functions	100	100
γ_L	Parameter for scaling L for P_o death	1	1
ν_{NG}	Uptake of nutrient by P_g cells	10	10
ν_{NO}	Uptake of nutrient by P_o cells	10	10
ν_{LO}	Uptake of L by P_o cells	10	10
$\overline{N_s}$	Parameter for nutrient source	2	2
W^*	Wnt level at which 50% of cells switch metabolism	5	5
N_d	Nutrient level below which cells will die	0.07	0.07
N_g^*	Nutrient level below which P_o cells cannot switch to glycolysis	0.1	0.1
β_N	Nutrient boundary condition parameter	0.25	0.25
S_x	Horizontal length of spatial domain	12	12
S_y	Vertical length of spatial domain	12	12
r	Parameter for scaling N for P_o death	0.375	0.375

A7.4 Simulations of DCA and XAV939 treatment on in vitro tumors

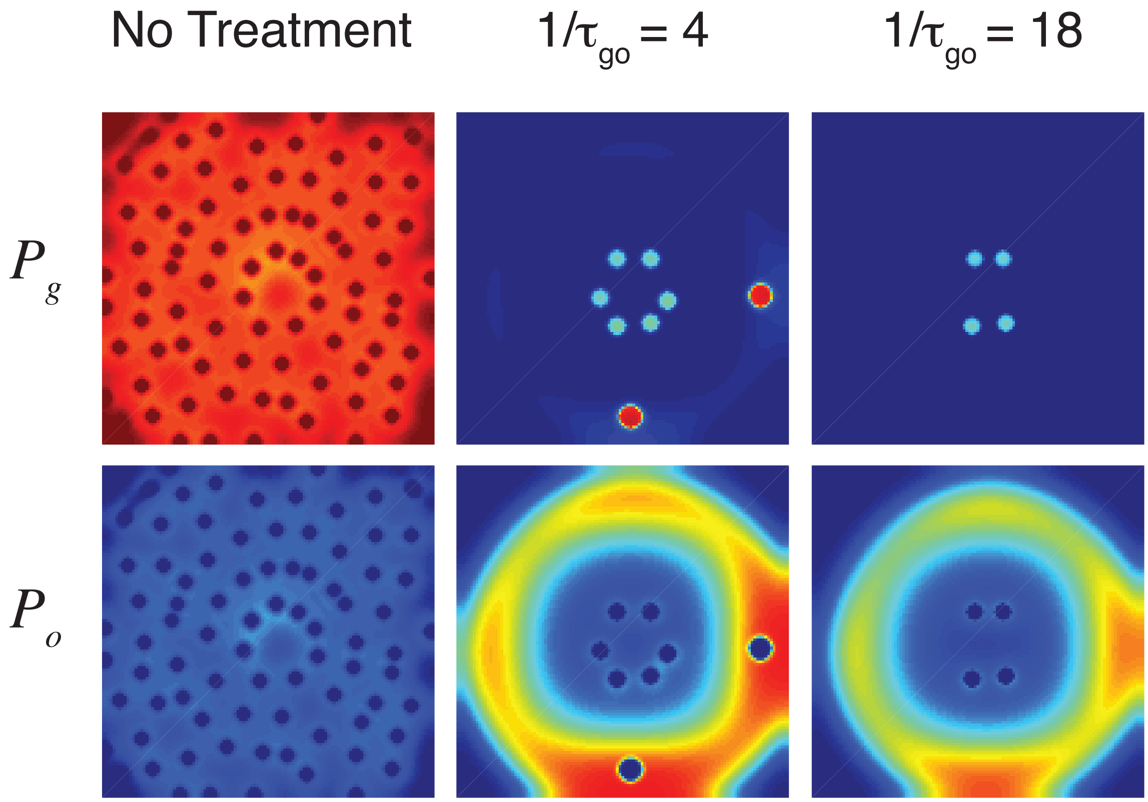

Appendix Figure S 17: Using the in vitro simulation equations described above, we modeled the same drug concentrations as the in vivo simulations presented in Figure 6. The tumor size over increasing drug concentrations for these simulations are described in Figure 7D.

A8 Synergy is evident in simulations with combined DCA and XAV939 treatment

We can measure the synergistic effect of treatment using the Bliss Independence model (Foucquier and Guedj, 2015). The Bliss combination index is defined by $BCI = \frac{E_A+E_B-E_AE_B}{E_{AB}}$, where the effectiveness of treatment by drug A is E_A and the effectiveness of treatment by drug B is E_B. The Bliss model assumes that the drugs A and B act independently of each other. Effectiveness for each drug is defined by the ability to reduce tumor size as in the following:

$$E_A = 1 - (\text{surviving tumor fraction after applying drug A})$$
$$E_B = 1 - (\text{surviving tumor fraction after applying drug B})$$
$$E_{AB} = 1 - (\text{sirviving tumor fraction after applying drug A and drug B in combination}).$$

A BCI of less than 1 indicates the synergy of drug action, while a score closer to 1 indicates the treatments act additively rather than synergistically. A BCI greater than 1 implies that the drugs interfere with each other. In the in vivo simulation and in vitro experimental model of tumor spheroid growth, $E_{\text{XAV939}} = E_{\text{DCA}} = 0$ (see figure below), which implies that BCI=0 in these cases.

In vivo:

Neither DCA alone nor XAV939 alone are successful in eradicating any of the tumor cells by the end of treatment (unitless time 50) unless the treatment doses are relatively high (e.g., $\bar{S}_W \lesssim 0.4$ or $1/\tau \gtrsim 30$). Thus, $E_{\text{XAV939}} = E_{\text{DCA}} = 0 = BCI$. In figure 18 below, we plot E_{AB} with A = XAV939 and B = DCA. The areas with dark blue indicate the regions where the combination effectiveness $E_{AB} = 1$ and the Bliss model predicts that there is synergy (this would also be true if we used the Highest Single Agent or Response Additivity models to assess synergy).

In vitro:

In the in vitro model simulations, we assessed synergy using $\overline{S_W} = 0.8$ (the same conditions for the red curve in figure 7D in the main text) and $\frac{1}{\tau_{go}} = 4$. We obtain $E_{\text{XAV949}} = 0.0156$, $E_{\text{DCA}} = 0.1855$, and $E_{\text{XAV949+DCA}} = 0.5725$. Thus $BCI = 0.3462$. The BCI depends on the drug concentrations, however. For example, taking $\overline{S_W} = 0.8$ and $\frac{1}{\tau_{go}} = 18$, we find $E_{\text{XAV949}} = .0156$ as before but now $E_{\text{DCA}} = 0.6679$, and $E_{\text{XAV949+DCA}} = 0.68$, which yields $BCI = 0.9898$. Thus, the BCI indicates that DCA and XAV939 combined treatments are more synergistic at smaller concentrations of DCA (the effect of DCA reaches a saturation point - see figure 7D in the main text).

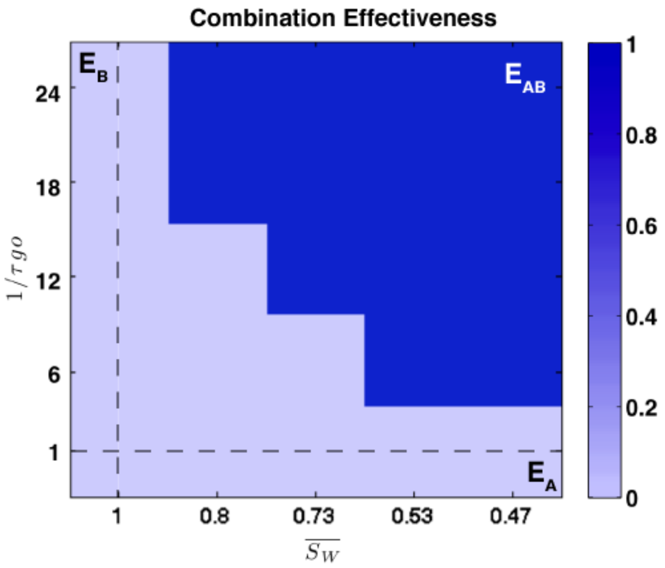

Appendix Figure S 18: Effectiveness of combination therapy. Here we plot the proportion of total tumor that is eradicated by DCA (vertical axis, $1/\tau_{go}$) and XAV939 (horizontal axis, $\overline{S_W}$) treatment, where 0 (light blue) means none of the tumor cells are killed by treatment, and 1 (dark blue) means 100% of the tumor cells have died. Pure DCA treatment (bottom row, $1/\tau_{go} = 1$) or pure XAV939 treatment (left column, $\overline{S_W} = 1$) are not at all effective in killing the tumor. Away from the bottom row and left column (as indicated by the dotted lines), the plot shows E_{AB} with $A =$ XAV949 and $B =$ DCA. The upper right area of the plot, which is a combination of low levels of DCA and XAV939 treatment together, shows that the tumor is completely killed, and hence a synergy between the two drugs.

A9 Heterogeneity in orthotopic tumors

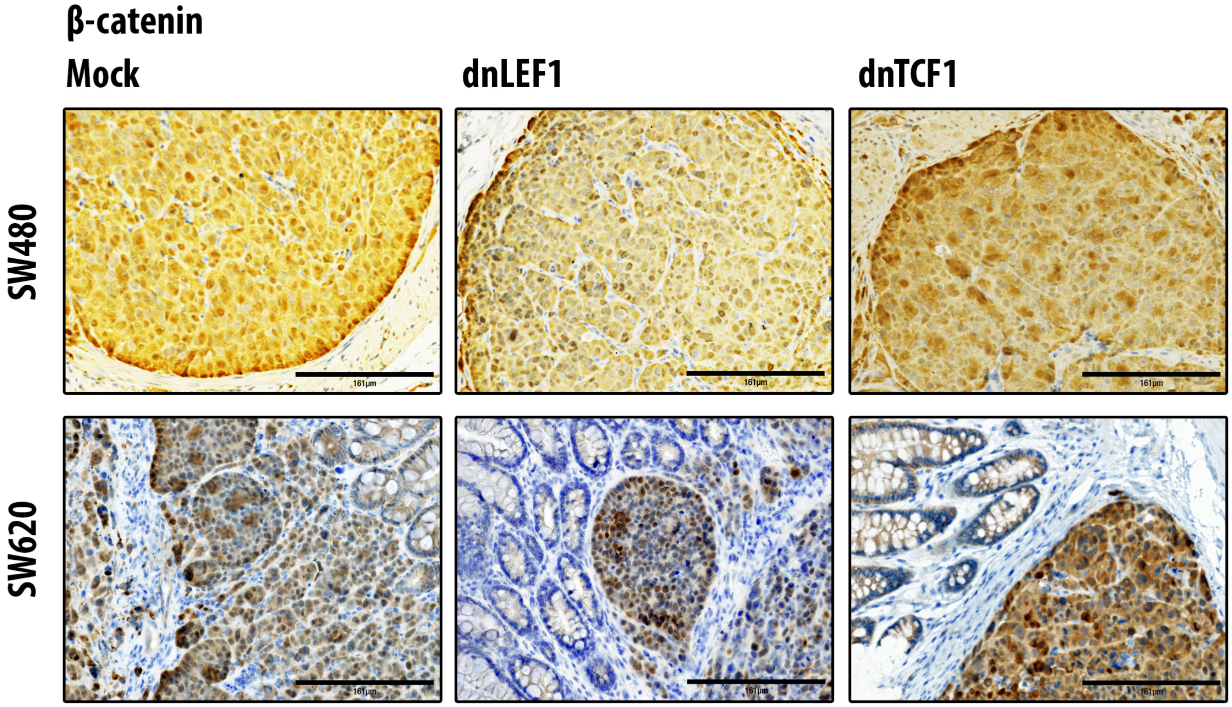

Appendix Figure S 19: β-catenin stains in SW480 and SW620 orthotopic tumors. Patterns of Wnt signaling heterogeneity are present in orthotopic tumors of SW480 and SW620 cells. Cell lines were lentivirally transduced to express dominant negative LEF1 or TCF1 and the transduced cells were injected into the submucosal layer of the colon wall. Tumors were harvested after three weeks and stained for beta-catenin. Scalebars are 161 um. Decreasing Wnt signaling in SW480 tumors leads to changes in hypoxic patterning. SW480 cells lentivirally transduced to express either an empty vector or dominant negative LEF1 were subcutaneously injected into immunocompromised mice. Tumor sections were stained for HIF1α.

A10 Vessel density versus pPDH expression in relative to Mock tumor

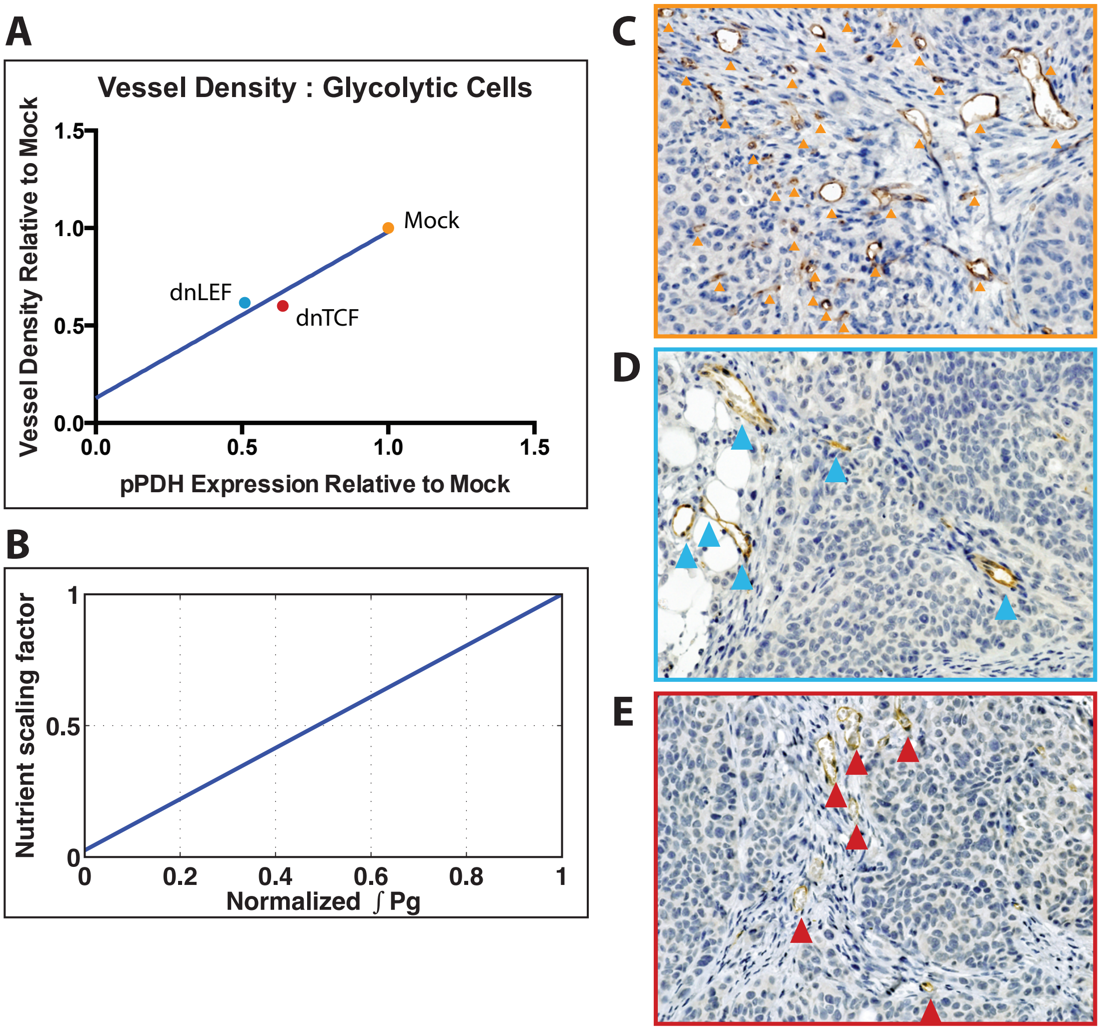

Appendix Figure S 20: We quantified the vasculature of the tumors by staining for CD31, an endothelial cell marker. We graphed the vessel density per field and the level of pPDH expression on the x-axis, both values normalized to Mock tumors. Mock tumors demonstrated significantly higher pPDH levels and vasculature than dnLEF or dnTCF tumors. Our mathematical model was capable of replicating the best fit line from the biological data.
