# Supplementary material for: Mathematical modeling links Wnt signaling to emergent patterns of metabolism in colon cancer
Source: Mol Syst Biol. 2017 Feb 9;13(2):912. doi: 10.15252/msb.20167386 (PMC5327728; doi:10.15252/msb.20167386)
Supplement: Supplementary file 2 — Expanded View Figures PDF [file MSB-13-912-s002.pdf]

Expanded View Figures

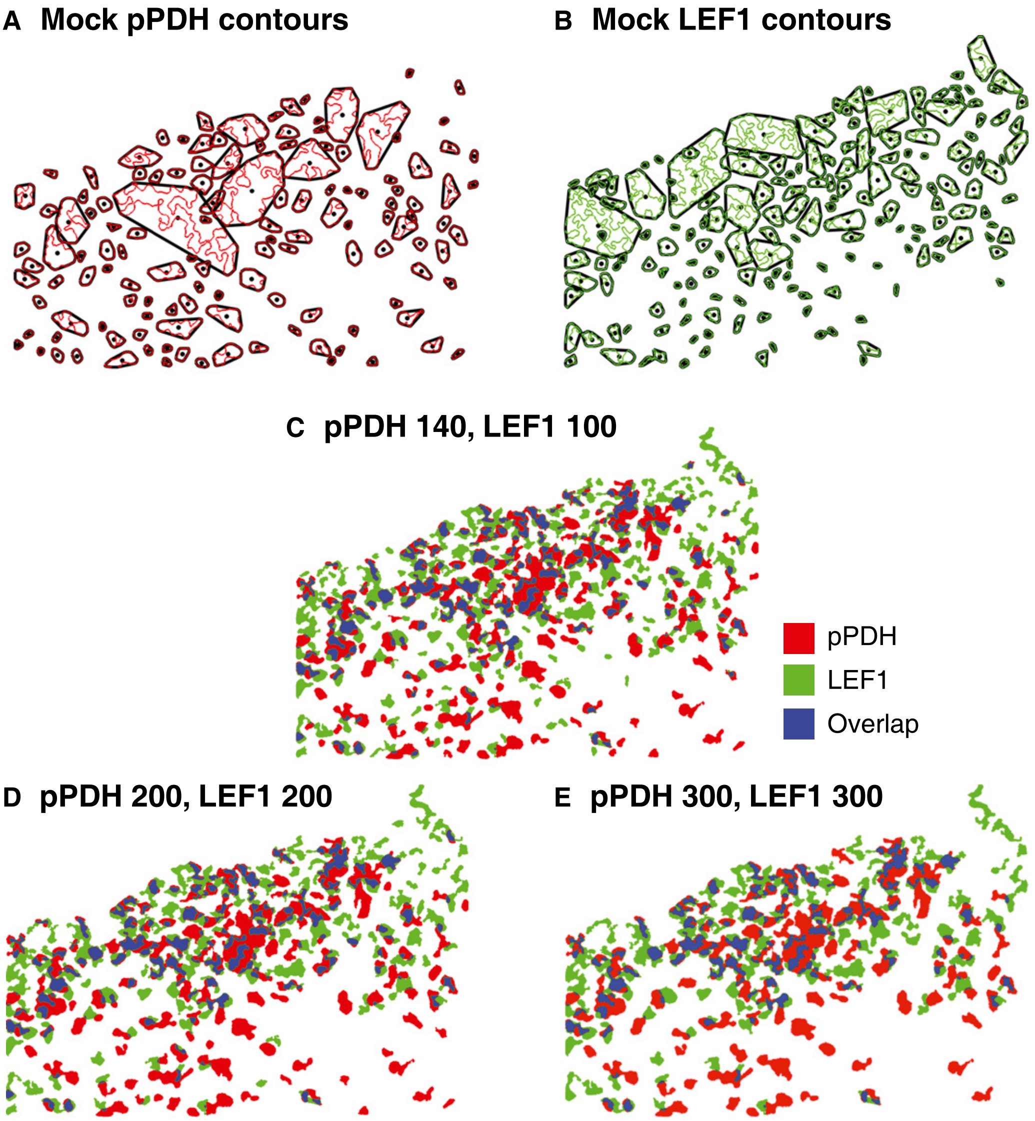

F

Threshold pPDH	Threshold LEF1	pPDH # of spots	pPDH # over-lapping	pPDH %	LEF1 # of spots	LEF1 # over-lapping	LEF1 %
140	100	131	99	75.57%	173	114	65.90%
200	200	108	79	73.15%	111	81	72.97%
300	300	80	59	73.75%	84	65	77.38%

Figure EV1. Overlap of pPDH and LEF1 spots in xenograft tumors.
Convex hull image analysis of serial sections of SW480 mock xenograft tumors stained for pPDH and LEF1, as shown in Fig 1A and B, second panels.

A, B Isolated contour maps with convex hull outlines for pPDH and LEF1.
C–E pPDH and LEF1 contour maps were overlaid on each other and overlapped regions highlighted in blue. Different thresholds for spot detection were tested; each threshold condition revealed between 65 and 77% overlap between pPDH and LEF1 spots.
F Summary of overlap results.

© 2017 The Authors

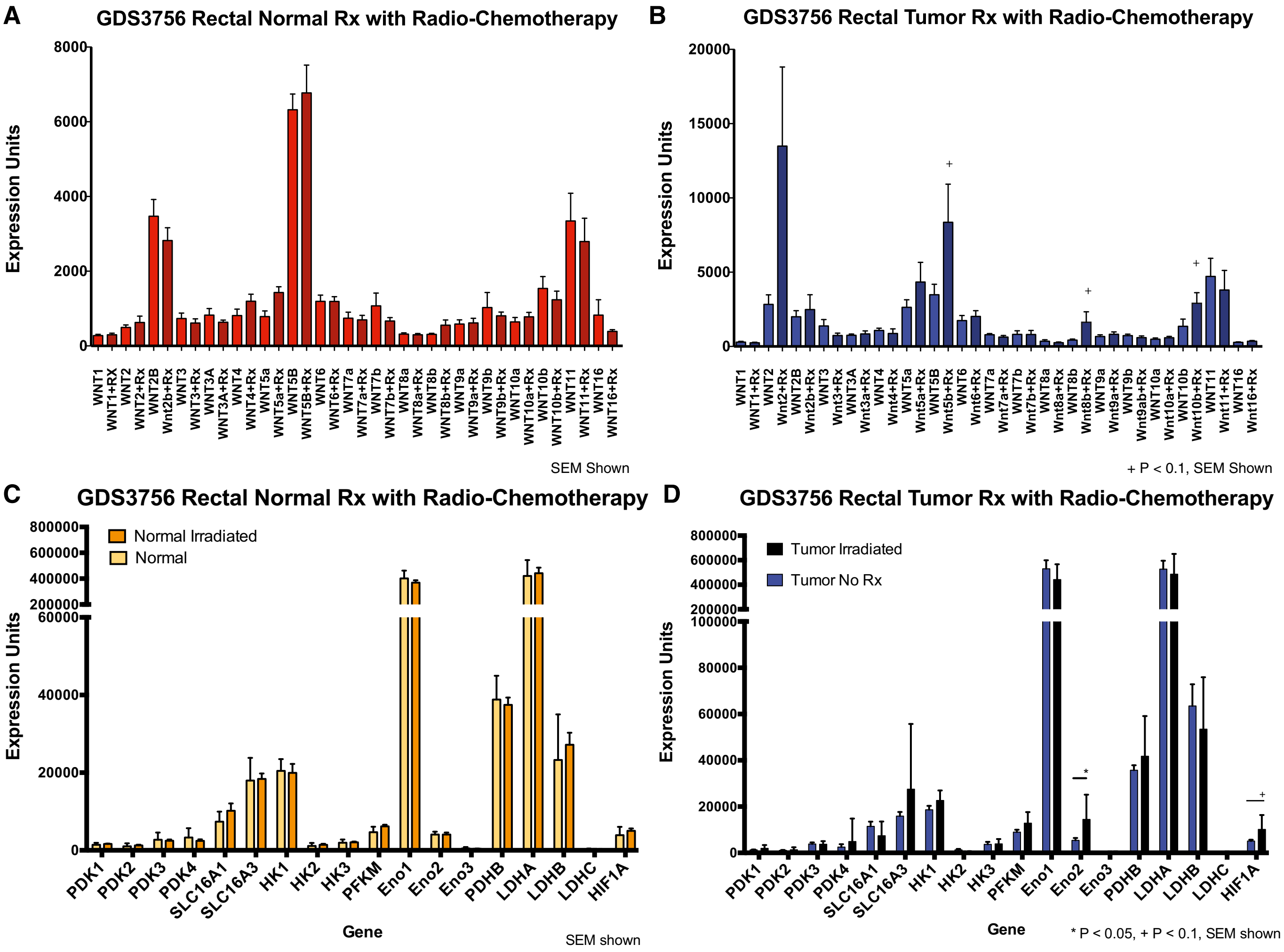

Figure EV2. Wnt ligand and glycolysis gene expression in rectal cancer patients post-radiochemotherapy.
Gene expression data, from GEO dataset GDS3756, of 21 rectal cancer patients before and after radio-chemotherapy (Snipstad *et al*, 2010).

A, B Expression of Wnt ligands WNT5B, WNT8B, and WNT10B shows trends toward increased expression in rectal tumor tissue treated with radiochemotherapy, but these changes do not reach statistical significance when $P < 0.05$ is used as a cutoff (specific *P*-values are 0.10, 0.10, and 0.08, respectively). Statistical significance was determined using the Mann–Whitney *U*-test with Benjamini–Hochberg correction for multiple hypothesis testing.

C, D Expression of the glycolytic enzyme ENO2 is specifically increased in tumor tissue after radiochemotherapy ($P = 0.008$); HIF1A expression also shows a trend in increased expression ($P = 0.06$). * denotes adjusted *P*-value < 0.05; + denotes adjusted *P*-value < 0.10. Statistical significance was determined using the Mann–Whitney *U*-test with Benjamini–Hochberg correction for multiple hypothesis testing.

Figure EV3. Targeted therapy simulations for P_g and P_o populations.
Figure 5 gives the results for total tumor size after individually targeting either P_g or P_o cells with given treatment doses and treatment times. Here are the effects for the individual P_g and P_o populations in those targeted therapy simulations. Simulations suggest that the glycolytic cell population is a more sensitive drug target than the oxidative cell population. We target either P_o (left) or P_g (right) cells selectively, starting from a metabolically patterned state, for 2.5, 5, or 7.5 (arbitrary) time units, with a death rate between 0.25 and 1. After therapy is stopped, the cells are allowed to evolve according to the original model (Fig 2). The P_g and P_o cell populations, relative to their initial cell populations, are shown.

 © 2017 The Authors

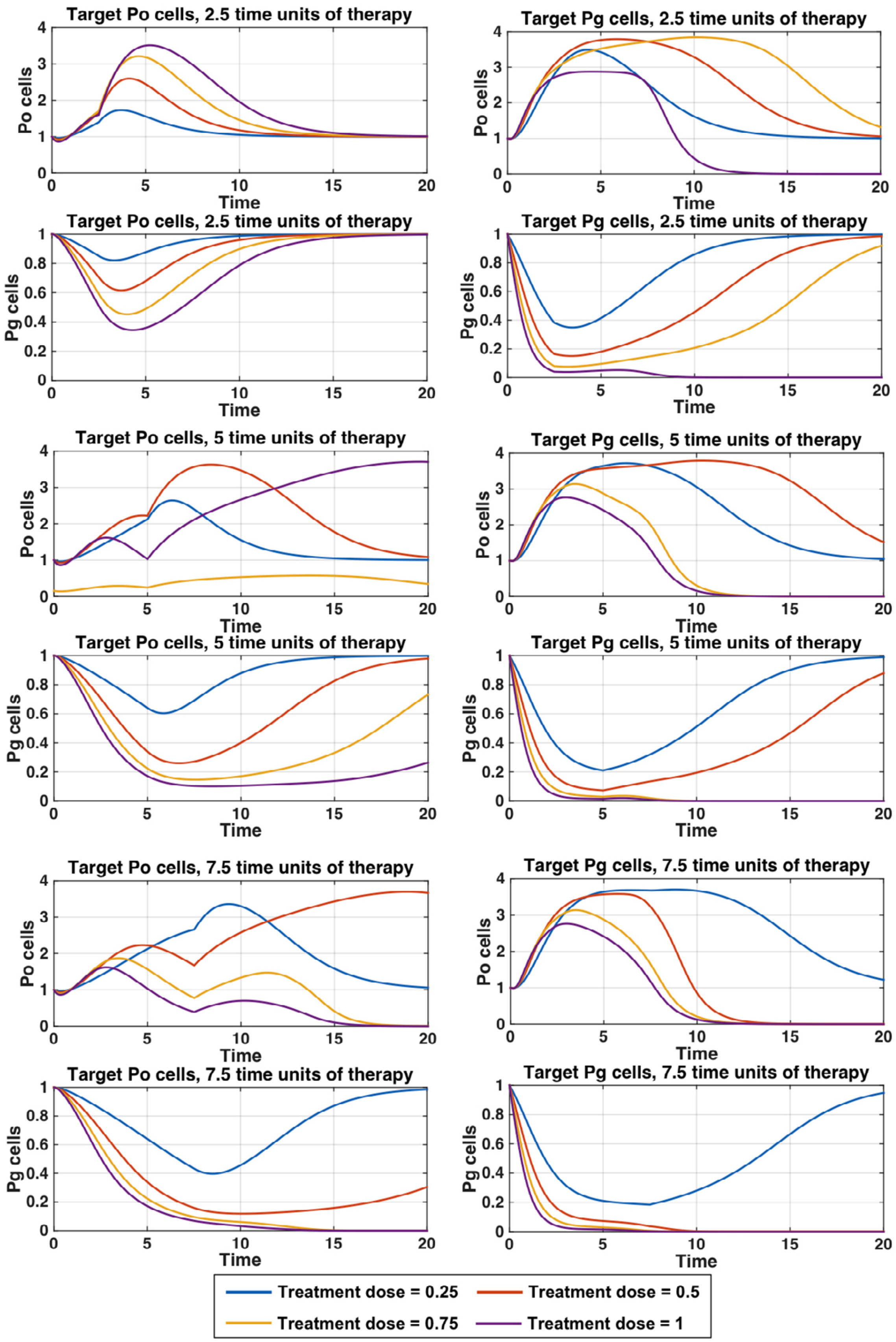

Figure EV3.

© 2017 The Authors
